# Dux4 controls migration of mesenchymal stem cells through the Cxcr4-Sdf1 axis

**DOI:** 10.18632/oncotarget.11368

**Published:** 2016-08-18

**Authors:** Petr Dmitriev, Ekaterina Kiseleva, Olga Kharchenko, Evgeny Ivashkin, Andrei Pichugin, Philippe Dessen, Thomas Robert, Frédérique Coppée, Alexandra Belayew, Gilles Carnac, Dalila Laoudj-Chenivesse, Marc Lipinski, Andrei Vasiliev, Yegor S. Vassetzky

**Affiliations:** ^1^ UMR 8126, Univ. Paris-Sud, CNRS, Institut de Cancérologie Gustave-Roussy, Villejuif, France; ^2^ LIA1066 Laboratoire Franco-Russe de Recherches en Oncologie, Villejuif, France; ^3^ N.K. Koltzov Institute of Developmental Biology, RAS, Moscow, Russia; ^4^ Functional Genomics Unit, Institut de Cancérologie Gustave-Roussy, Villejuif, France; ^5^ Laboratory of Molecular Biology, Research Institute for Health Sciences and Technology, University of Mons, Mons, Belgium; ^6^ PhyMedExp, University of Montpellier, INSERM U1046, CNRS UMR 9214, Montpellier, France; ^7^ Peter the Great St. Petersburg Polytechnic University, St. Petersburg, Russia

**Keywords:** DUX4, CXCR4, SDF1, signalling, migration

## Abstract

We performed transcriptome profiling of human immortalized myoblasts (MB) transiently expressing double homeobox transcription factor 4 (DUX4) and double homeobox transcription factor 4 centromeric (DUX4c) and identified 114 and 70 genes differentially expressed in *DUX4-* and *DUX4c-*transfected myoblasts, respectively. A significant number of differentially expressed genes were involved in inflammation, cellular migration and chemotaxis suggesting a role for DUX4 and DUX4c in these processes. *DUX4* but not *DUX4c* overexpression resulted in upregulation of the CXCR4 (C-X-C motif Receptor 4) and CXCL12 (C-X-C motif ligand 12 also known as SDF1) expression in human immortalized myoblasts. In a Transwell cell migration assay, human bone marrow-derived mesenchymal stem cells (BMSCs) were migrating more efficiently towards human immortalized myoblasts overexpressing DUX4 as compared to controls; the migration efficiency of DUX4-transfected BMSCs was also increased. DUX4c overexpression in myoblasts or in BMSCs had no impact on the rate of BMSC migration. Antibodies against SDF1 and CXCR4 blocked the positive effect of DUX4 overexpression on BMSC migration. We propose that DUX4 controls the cellular migration of mesenchymal stem cells through the CXCR4 receptor.

## INTRODUCTION

Human genome harbors 333 genes and pseudogenes with homeobox sequences encoding homeodomain DNA binding motif. Most of homeobox genes are transcription factors of which many are known to play key roles in animal development (for review see [1]). Among all homeobox genes, the double homeobox (DUX) family is one of the most enigmatic. DUX family members numbered from 1 to 5 [2–4] are encoded within tandem repeats of macrosatellite DNA and their multiple polymorphic copies are spread over human genome. Two other members of the DUX family, DUXA [5] and DUXB [6] are single-copy genes encoded on chromosome 19 and 16, respectively.

Double homeobox protein 4 (DUX4) and its nearly identical homologue DUX4 centromeric (DUX4c) are the best known of all DUX proteins (for review see [7]). Multiple copies of *DUX4* ORFs reside within 3.3 kb-long macrosatellite repeats on chromosomes 4q35 and 10q26 also called D4Z4 [8, 3]. The copy number of *DUX4* ORF may vary from several units to several hundred making it the highest copy number ORF in human genome [9]. The single-copy of *DUX4c* gene is located 42 kb proximally to D4Z4 array on chromosome 4q35 [10].

The alternative splicing of *DUX4* pre-mRNA results in the production of either a full-length 424 amino acid-long or truncated 160-aa protein lacking the C-terminal transactivation domain (DUX4-s) [11–12]. High level of full-length *DUX4* overexpression was shown to be toxic for mouse and human cultured cells [13–16]. *In vivo*, ubiquitous *DUX4* overexpression is detrimental for zebrafish [17] and *Xenopus* [18] development. Muscle-specific *DUX4* overexpression resulted in tissue deterioration [19, 18, 20] specific overexpression in other tissues types was not tested. DUX4 toxicity has been linked to p53-dependent apoptosis induction [19, 13, 14, 19] and has been shown to require the C terminus [15] and the integrity of DNA binding domains [19, 15] of DUX4. Other biological effects of *DUX4* overexpression *in vitro* include an increased sensitivity to oxidative stress and an inhibition of myogenic differentiation of human and mouse myogenic progenitor cells [14, 21].

In contrast to DUX4, high level of *DUX4c* [22, 23] or *DUX4-s* [24] expression is not toxic for the cells in culture. *DUX4c* overexpression induced human myoblast proliferation [22] and inhibited myogenic differentiation [23]; phenotypic effects of *DUX4-s* overexpression were not described, however it has been shown to inhibit DUX4 target genes when overexpressed together with *DUX4* [12]. Neither ubiquitous nor muscle-specific *DUX4c* expression *in vivo* interfered with embryogenesis or muscle tissue integrity in *Xenopus* [18]. Similarly, *DUX4-s* injection did not affect normal development of zebrafish embryos [20].

To better understand the mechanism of DUX4 impact on the cell, we performed transcriptome profiling of human primary myoblasts overexpressing full-length *DUX4* and *DUX4c*. One of the most striking findings of this analysis was an apparent upregulation of a significant number of chemokine genes. Chemokines is a superfamily of vertebrate-specific protein ligands interacting with rhodopsin-like G-protein-coupled receptors (GPCRs) [25]. The primary role of chemokines is the control of leukocyte traffic and recruitment to inflamed tissues (for review see [26–30]). Some of constitutively produced chemokines are known to play a role in processes unrelated to innate or acquired immunity (for review see [31–32]). Of these, CXCL12 (C-X-C motif ligand 12), also known as Stromal-derived factor 1 (SDF1) [33]) and its primary receptor CXCR4 (C-X-C motif Receptor 4) [34] clearly stand apart due to an important role in development regulation [35], hematopoiesis, angiogenesis [36–37], stem cell migration [38] and wound healing (reviewed in [39–40]). In addition, CXCR4 is one of the major GPCR receptors controlling the migration of various cell types including leukocytes, HSPC, MSC (reviewed in [41].

MSC (mesenchymal stem or mesenchymal stromal cells) are multipotent stem cells capable of differentiation into chondrocytes, osteocytes, adipocytes and, more controversially, to other cell types (for review see [42]). The primary site of MSC localization is bone marrow, although cells with similar properties were also found in other tissues (for review see [43]). In adult organism, MSC are thought to contribute to tissue homeostasis. Tissue damage results in MSC mobilization from bone marrow and their homing to damaged tissue where they either differentiate to replenish damaged cells, stimulate differentiation of the tissue-resident stem cells, stimulate vascularization and control inflammation thus restoring the damage (reviewed in [44]). CXCR4-SDF1 signaling is known to be required for MSC homing and retention to their niche in bone marrow (for review see [43]) and has also been shown to stimulate MSC cell migration *in vitro* [45, 46]. We have shown that *DUX4* overexpression results in upregulation of *CXCR4* and *SDF1* genes in several cell types and stimulates the migration of BMSC in a CXCR4- and SDF1-dependent manner. Our results thus establish DUX4 as a novel regulator of cell mobility.

## RESULTS

### Transcriptome profiling of *DUX4*- and *DUX4c*-overexpressing human myoblasts

**Figure 1:**
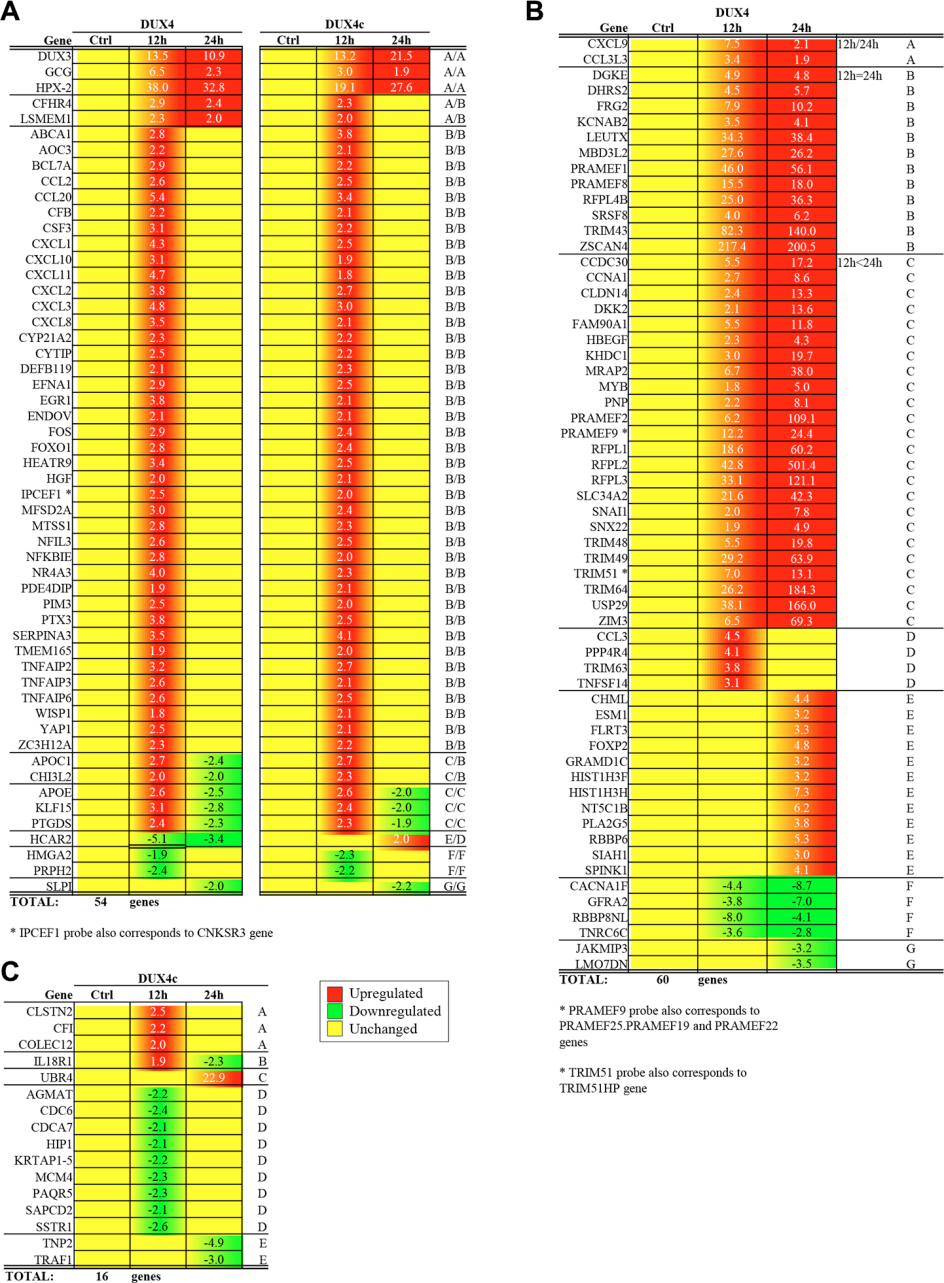
Genes differentially expressed 12- and 24 h after the transfection of human immortalized myoblasts (MB) with DUX4 or DUX4c plasmids as compared to empty vector; yellow: no differential expression (-1.5 < FC < 1.5); red: upregulated (FC > 1.5); green: downregulated (FC < −1.5) (**A**) Genes differentially expressed in both DUX4- and DUX4c-transfected MB; genes upregulated at both 12 h and 24 h, only at 12 h or 24 h time-points are labeled with (A, B and D) respectively; genes upregulated at 12 h but downregulated at 24 h time-point are labeled with C; genes downregulated at both 12 h and 24 h, only at 12 h or 24 h time-points are labeled with (E, F and G) respectively. (**B**) Genes differentially expressed only in DUX4-transfected MB; genes upregulated at both 12 h and 24 h time-points with the expression level at 12 h higher, equal or lower than at 24 h are labeled with (A, B or C) respectively; genes upregulated only at 12 h or 24 h time-points are labeled with D and E respectively; genes downregulated at both time-points or only at 24 h time-point are labeled with F and G respectively. (**C**) Genes differentially expressed only in DUX4c-transfected MB; genes upregulated at 12 h or 24 h or downregulated at 12 h or 24 h time-points are labeled with A, C, D and E respectively; genes upregulated at 12 h but downregulated at 24 h time-points are labeled with B.

Several transcriptomic studies have been performed on cells overexpressing *DUX4*, at the same time its homologue *DUX4c* is much less studied. We argued that the difference in transcriptome profiles of *DUX4* and *DUX4c* might help us to better understand the functional differences between these two proteins. We thus performed transcriptome profiling of *DUX4-* and *DUX4c-* transfected immortalized human myoblasts (MB) at two time points (12 and 24 h) following transfection. Overall, we have identified 130 differentially expressed genes of which 60 genes were differentially expressed only in *DUX4-*transfected cells, 16 genes only in *DUX4c* transfected cells and 54 genes were differentially expressed both in *DUX4-* and *DUX4c-*transfected cells (Figure 1). With rare exceptions, all significantly overexpressed DUX4 target genes found in our study were already described previously [12] (Tables 1–4 and Supplementary Table S1). DUX4 is known to induce apoptosis in various cell types including human myoblasts. Indeed, we observed a significant induction of apoptosis and cell mortality in *DUX4*-transfected MB 72 h after the transfection (data not shown). However, 24 h after the transfection, apoptosis and cell mortality were significantly increased neither in *DUX4-* nor in *DUX4c-*transfected MB indicating that our transcriptomic data obtained at earlier time points may contain information about other functional activities of DUX4 (Supplementary Figure S1).

**Table 1:** Genes differentially expressed in hIMB cells 12 h after DUX4-plasmid transfection

DUX4 12 h				
	FC	Gene	Description	ref
	217.4	ZSCAN4	zinc finger and SCAN domain containing 4	^‡^
	82.3	TRIM43	tripartite motif containing 43	^‡^
	46.0	PRAMEF1	PRAME family member 1	^‡^
	42.8	RFPL2	ret finger protein-like 2	^‡^
	38.1	USP29	ubiquitin specific peptidase 29	^‡^
*	38.0	HPX-2	homeobox HPX-2	
	34.3	LEUTX	leucine twenty homeobox	
	33.1	RFPL3	ret finger protein-like 3	^‡^
	29.2	TRIM49	tripartite motif containing 49	^‡^
	27.6	MBD3L2	methyl-CpG binding domain protein 3-like 2	^‡^
	26.2	TRIM64	tripartite motif containing 64	^‡^
	25.0	RFPL4B	ret finger protein-like 4B	^‡^
	21.6	SLC34A2	solute carrier family 34 (type II sodium/phosphate contransporter). member 2	^‡^
	18.6	RFPL1	ret finger protein-like 1	^‡^
	15.5	PRAMEF8	PRAME family member 8	^‡^
*	13.5	DUX3	double homeobox 3	
	12.2	PRAMEF9; PRAMEF25; PRAMEF19; PRAMEF22	PRAME family member 9;PRAME family member 25;PRAME family member 19;PRAME family member 22	^‡^
	7.9	FRG2	FSHD region gene 2	
	7.5	CXCL9	chemokine (C-X-C motif) ligand 9	
	7.0	TRIM51. TRIM51HP	tripartite motif-containing 51;tripartite motif-containing 51H. pseudogene	
	6.7	MRAP2	melanocortin 2 receptor accessory protein 2	
*	6.5	GCG	glucagon	
	6.5	ZIM3	zinc finger. imprinted 3	
	6.2	PRAMEF2	PRAME family member 2	^‡^
	5.5	FAM90A1	family with sequence similarity 90. member A1	^‡^
	5.5	TRIM48	tripartite motif containing 48	^‡^
	5.5	CCDC30	coiled-coil domain containing 30	
*	5.4	CCL20	chemokine (C-C motif) ligand 20	
	4.9	DGKE	diacylglycerol kinase. epsilon 64kDa	
*	4.8	CXCL3	chemokine (C-X-C motif) ligand 3	
*	4.7	CXCL11	chemokine (C-X-C motif) ligand 11	
	4.5	CCL3	chemokine (C-C motif) ligand 3	
	4.5	DHRS2	dehydrogenase/reductase (SDR family) member 2	
*	4.3	CXCL1	chemokine (C-X-C motif) ligand 1 (melanoma growth stimulating activity. alpha)	
	4.1	PPP4R4	protein phosphatase 4. regulatory subunit 4	
*	4.0	NR4A3	nuclear receptor subfamily 4. group A. member 3	
	4.0	SRSF8	serine/arginine-rich splicing factor 8	
	3.8	TRIM63	tripartite motif containing 63. E3 ubiquitin protein ligase	
*	3.8	CXCL2	chemokine (C-X-C motif) ligand 2	
*	3.8	PTX3	pentraxin 3. long	
*	3.8	EGR1	early growth response 1	
*	3.5	CXCL8	chemokine (C-X-C motif) ligand 8	
*	3.5	SERPINA3	serpin peptidase inhibitor. clade A (alpha-1 antiproteinase. antitrypsin). member 3	
	3.5	KCNAB2	potassium voltage-gated channel. shaker-related subfamily. beta member 2	
	3.4	CCL3L3	chemokine (C-C motif) ligand 3-like 3	
*	3.4	HEATR9	HEAT repeat containing 9	
*	3.2	TNFAIP2	tumor necrosis factor. alpha-induced protein 2	
	3.1	TNFSF14	tumor necrosis factor (ligand) superfamily. member 14	
*	3.1	KLF15	Kruppel-like factor 15	
*	3.1	CXCL10	chemokine (C-X-C motif) ligand 10	
*	3.1	CSF3	colony stimulating factor 3 (granulocyte)	
	...	...	...	
	–3.6	TNRC6C	trinucleotide repeat containing 6C	
	–3.8	GFRA2	GDNF family receptor alpha 2	
	–4.4	CACNA1F	calcium channel. voltage-dependent. L type. alpha 1F subunit	
*	–5.1	HCAR2	hydroxycarboxylic acid receptor 2	
	–8.0	RBBP8NL	RBBP8 N-terminal like	

Asterisk labels genes that are also differentially expressed in DUX4c-transfected IMBs.

‡ labels genes previously descried DUX4 target genes [12].

**Table 2:** Genes differentially expressed in hIMB cells 24 h after DUX4-plasmid transfection

DUX4 24 h				
	FC	Gene	Description	ref
	501.4	RFPL2	ret finger protein-like 2	‡
	200.5	ZSCAN4	zinc finger and SCAN domain containing 4	‡
	184.3	TRIM64	tripartite motif containing 64	‡
	166.0	USP29	ubiquitin specific peptidase 29	‡
	140.0	TRIM43	tripartite motif containing 43	‡
	121.1	RFPL3	ret finger protein-like 3	‡
	109.1	PRAMEF2	PRAME family member 2	‡
	69.3	ZIM3	zinc finger. imprinted 3	
	63.9	TRIM49	tripartite motif containing 49	‡
	60.2	RFPL1	ret finger protein-like 1	‡
	56.1	PRAMEF1	PRAME family member 1	‡
	42.3	SLC34A2	solute carrier family 34 (type II sodium/phosphate contransporter). member 2	‡
	38.4	LEUTX	leucine twenty homeobox	
	38.0	MRAP2	melanocortin 2 receptor accessory protein 2	
	36.3	RFPL4B	ret finger protein-like 4B	‡
*	32.8	HPX-2	homeobox HPX-2	
	26.2	MBD3L2	methyl-CpG binding domain protein 3-like 2	‡
	24.4	PRAMEF9. PRAMEF25. PRAMEF19. PRAMEF22	PRAME family member 9;PRAME family member 25;PRAME family member 19;PRAME family member 22	‡
	19.8	TRIM48	tripartite motif containing 48	‡
	19.7	KHDC1	KH homology domain containing 1	‡
	18.0	PRAMEF8	PRAME family member 8	‡
	17.2	CCDC30	coiled-coil domain containing 30	
	13.6	DKK2	dickkopf WNT signaling pathway inhibitor 2	
	13.3	CLDN14	claudin 14	
	13.1	TRIM51. TRIM51HP	tripartite motif-containing 51;tripartite motif-containing 51H. pseudogene	
	11.8	FAM90A1	family with sequence similarity 90. member A1	‡
*	10.9	DUX3	double homeobox 3	
	10.24	FRG2	FSHD region gene 2	
	8.61	CCNA1	cyclin A1	
	8.14	PNP	purine nucleoside phosphorylase	
	7.75	SNAI1	snail family zinc finger 1	
	7.34	HIST1H3H	histone cluster 1. H3h	
	6.19	NT5C1B	5′-nucleotidase. cytosolic IB	
	6.17	SRSF8	serine/arginine-rich splicing factor 8	
	5.69	DHRS2	dehydrogenase/reductase (SDR family) member 2	
	5.33	RBBP6	retinoblastoma binding protein 6	
	4.96	MYB	v-myb avian myeloblastosis viral oncogene homolog	
	4.92	SNX22	sorting nexin 22	
	4.79	FOXP2	forkhead box P2	
	4.78	DGKE	diacylglycerol kinase. epsilon 64kDa	
	4.37	CHML	choroideremia-like (Rab escort protein 2)	
	4.32	HBEGF	heparin-binding EGF-like growth factor	
	4.14	KCNAB2	potassium voltage-gated channel. shaker-related subfamily. beta member 2	
	4.10	SPINK1	serine peptidase inhibitor. Kazal type 1	
	3.84	PLA2G5	phospholipase A2. group V	
	3.30	FLRT3	fibronectin leucine rich transmembrane protein 3	
	3.22	HIST1H3F	histone cluster 1. H3f	
	3.20	ESM1	endothelial cell-specific molecule 1	
	3.17	GRAMD1C	GRAM domain containing 1C	
	3.01	SIAH1	siah E3 ubiquitin protein ligase 1	‡
	...	...	...	
	–3.19	JAKMIP3	Janus kinase and microtubule interacting protein 3	
*	–3.36	HCAR2	hydroxycarboxylic acid receptor 2	
	–3.51	LMO7DN	LMO7 downstream neighbor	
	–4.10	RBBP8NL	RBBP8 N-terminal like	
	–7.00	GFRA2	GDNF family receptor alpha 2	
	–8.72	CACNA1F	calcium channel. voltage-dependent. L type. alpha 1F subunit	

Asterisk labels genes that are also differentially expressed in DUX4c-transfected IMBs.

**Table 3:** Genes differentially expressed in hIMB cells 12 h after DUX4c-plasmid transfection

DUX4c 12 h			
	FC	Gene	Description
*	19.1	HPX-2	homeobox HPX-2
*	13.2	DUX3	double homeobox 3
*	4.1	SERPINA3	serpin peptidase inhibitor. clade A (alpha-1 antiproteinase. antitrypsin). member 3
*	3.8	ABCA1	ATP-binding cassette. sub-family A (ABC1). member 1
*	3.4	CCL20	chemokine (C-C motif) ligand 20
*	3.0	GCG	glucagon
*	3.0	CXCL3	chemokine (C-X-C motif) ligand 3
*	2.7	CXCL2	chemokine (C-X-C motif) ligand 2
*	2.7	TNFAIP2	tumor necrosis factor. alpha-induced protein 2
*	2.7	APOC1	apolipoprotein C-I
*	2.6	APOE	apolipoprotein E
*	2.6	MFSD2A	major facilitator superfamily domain containing 2A
	2.5	CLSTN2	calsyntenin 2
*	2.5	HEATR9	HEAT repeat containing 9
*	2.5	EFNA1	ephrin-A1
*	2.5	CCL2	chemokine (C-C motif) ligand 2
*	2.5	CXCL1	chemokine (C-X-C motif) ligand 1 (melanoma growth stimulating activity. alpha)
*	2.5	TNFAIP6	tumor necrosis factor. alpha-induced protein 6
*	2.5	NFIL3	nuclear factor. interleukin 3 regulated
*	2.5	PTX3	pentraxin 3. long
*	2.4	FOXO1	forkhead box O1
*	2.4	KLF15	Kruppel-like factor 15
*	2.4	FOS	FBJ murine osteosarcoma viral oncogene homolog
*	2.3	NR4A3	nuclear receptor subfamily 4. group A. member 3
*	2.3	CHI3L2	chitinase 3-like 2
*	2.3	MTSS1	metastasis suppressor 1
*	2.3	DEFB119	defensin. beta 119
*	2.3	CFHR4	complement factor H-related 4
*	2.3	PTGDS	prostaglandin D2 synthase 21kDa (brain)
*	2.2	CSF3	colony stimulating factor 3 (granulocyte)
*	2.2	CYTIP	cytohesin 1 interacting protein
*	2.2	BCL7A	B-cell CLL/lymphoma 7A
	2.2	CFI	complement factor I
*	2.2	CYP21A2	cytochrome P450. family 21. subfamily A. polypeptide 2
*	2.2	ZC3H12A	zinc finger CCCH-type containing 12A
*	2.1	CXCL8	chemokine (C-X-C motif) ligand 8
*	2.1	PDE4DIP	phosphodiesterase 4D interacting protein
*	2.1	CFB	complement factor B
*	2.1	HGF	hepatocyte growth factor (hepapoietin A; scatter factor)
*	2.1	WISP1	WNT1 inducible signaling pathway protein 1
*	2.1	EGR1	early growth response 1
*	2.1	YAP1	Yes-associated protein 1
	2.1	AOC3	amine oxidase. copper containing 3
*	2.1	TNFAIP3	tumor necrosis factor. alpha-induced protein 3
*	2.1	ENDOV	endonuclease V
*	2.0	PIM3	Pim-3 proto-oncogene. serine/threonine kinase
*	2.0	NFKBIE	nuclear factor of kappa light polypeptide gene enhancer in B-cells inhibitor. epsilon
*	2.0	IPCEF1; CNKSR3	interaction protein for cytohesin exchange factors 1; CNKSR family member 3
*	2.0	LSMEM1	leucine-rich single-pass membrane protein 1
*	2.0	TMEM165	transmembrane protein 165
	2.0	COLEC12	collectin sub-family member 12
	...	...	...
	– 2.1	HIP1	huntingtin interacting protein 1
	– 2.1	SAPCD2	suppressor APC domain containing 2
	– 2.1	CDCA7	cell division cycle associated 7
*	– 2.2	PRPH2	peripherin 2 (retinal degeneration. slow)
	– 2.2	AGMAT	agmatine ureohydrolase (agmatinase)
	– 2.2	KRTAP1-5	keratin associated protein 1-5
*	– 2.3	HMGA2	high mobility group AT-hook 2
	– 2.3	PAQR5	progestin and adipoQ receptor family member V
	– 2.3	MCM4	minichromosome maintenance complex component 4
	– 2.4	CDC6	cell division cycle 6
	– 2.6	SSTR1	somatostatin receptor 1

Asterisk labels genes that are also differentially expressed in DUX4-transfected IMBs.

**Table 4:** Genes differentially expressed in hIMB cells 20 h after DUX4c-plasmid transfection

DUX4c 24 h			
	FC	Gene	Description
*	27.6067	HPX-2	homeobox HPX-2
	22.8739	UBR4	ubiquitin protein ligase E3 component n-recognin 4
*	21.4539	DUX3	double homeobox 3
*	–2.0324	KLF15	Kruppel-like factor 15
*	–2.1708	SLPI	secretory leukocyte peptidase inhibitor
	–2.2869	IL18R1	interleukin 18 receptor 1
	–2.9995	TRAF1	TNF receptor-associated factor 1
	–4.9447	TNP2	transition protein 2 (during histone to protamine replacement)

Asterisk labels genes that are also differentially expressed in DUX4-transfected IMBs.

**Figure 2:**
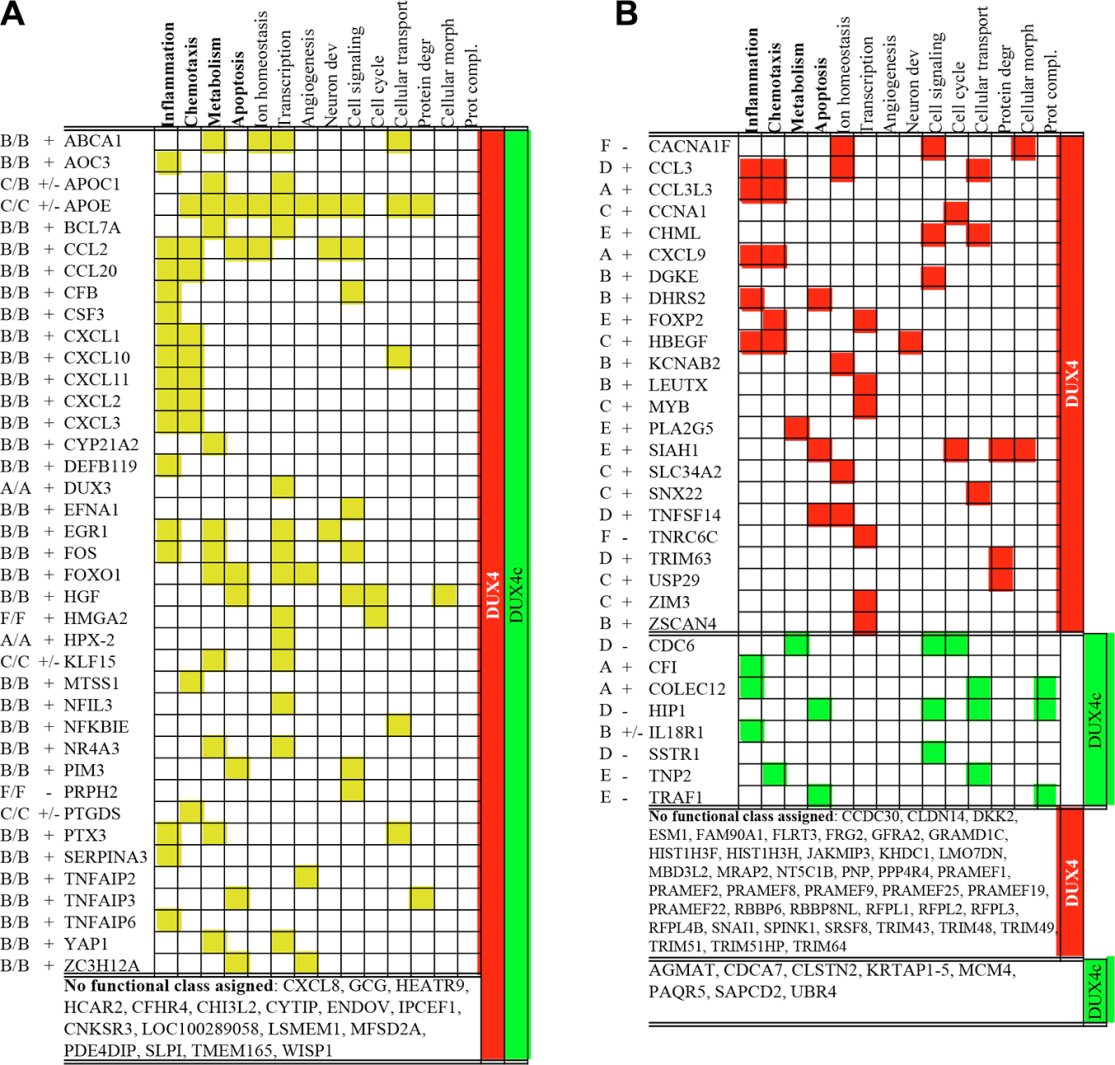
Functional classification of genes differentially expressed both in DUX4- and DUX4c- transfected human immortalized myoblasts (MB) (A) or only in DUX4- or DUX4c-transfected MB (B) at two time points (12- and 24 h) after the transfection Names of superclusters composed of several gene ontology functional categories (see supplementary Table S2 for the composition of superclusters) of which at least one was statistically significant (*p-value* < 0.05, FDR < 20) are in bold. Gene expression level at various time-points is indicated with letters (A–E or F) as in Figure 1; “+” and “−” indicate whether a gene was consistently up- or downregulated at all time points tested; “+/−” means that a gene was upregulated and one time-point but downregulated at the other.

According to GeneOntology analysis, significant known functions of the majority of genes differentially expressed in *DUX4* and *DUX4c*-overexpressing myoblasts included inflammation, chemotaxis, metabolism and apoptosis (Figure 2 and Supplementary Table S2). These functional categories have already been attributed to DUX4 target genes in previous transcriptomic studies of genes differentially expressed in *DUX4*-overexpressing murine C2C12 myoblasts [14], human primary myoblasts [12] and rhabdomyosarcoma cells [47]; furthermore, functional involvement of ectopically expressed *DUX4* in certain aspects of cellular metabolism and regulation of apoptosis has been previously demonstrated [13, 14].

Several functional categories including, transcription, angiogenesis, neuron development, cell signaling, cellular transport, protein degradation, and protein complex assembly that have not reach statistical significance in our study, were attributed to DUX4 target genes previously [14, 12, 47]. The validity of some of these functional categories has been experimentally proven: *DUX4* overexpression in human myoblasts has been shown to induce genes involved in protein degradation [16] and interfere with protein ubiquitination [48]. *DUX4* overexpression in mouse embryonic stem cells (ES) has been shown to induce neuroectoderm program [49]; the involvement of DUX4 in transcription regulation has also been addressed [14]. Finally, DUX4 target genes found in this study also include FRG2 and KLF15 - genes shown previously to be overexpressed in FSHD; this is in agreement with an observation that DUX4 target genes are differentially expressed in FSHD [50].

As compared to *DUX4c, DUX4* overexpression resulted in a differential expression of more genes involved in chemotaxis, ion transport, and protein degradation. Conversely, all differentially expressed genes related to protein complex assembly were exclusively controlled by *DUX4c* (Figure 2).

### *DUX4* overexpression results in upregulation of *CXCR4* and *SDF1* gene expression in various cell types

**Figure 3:**
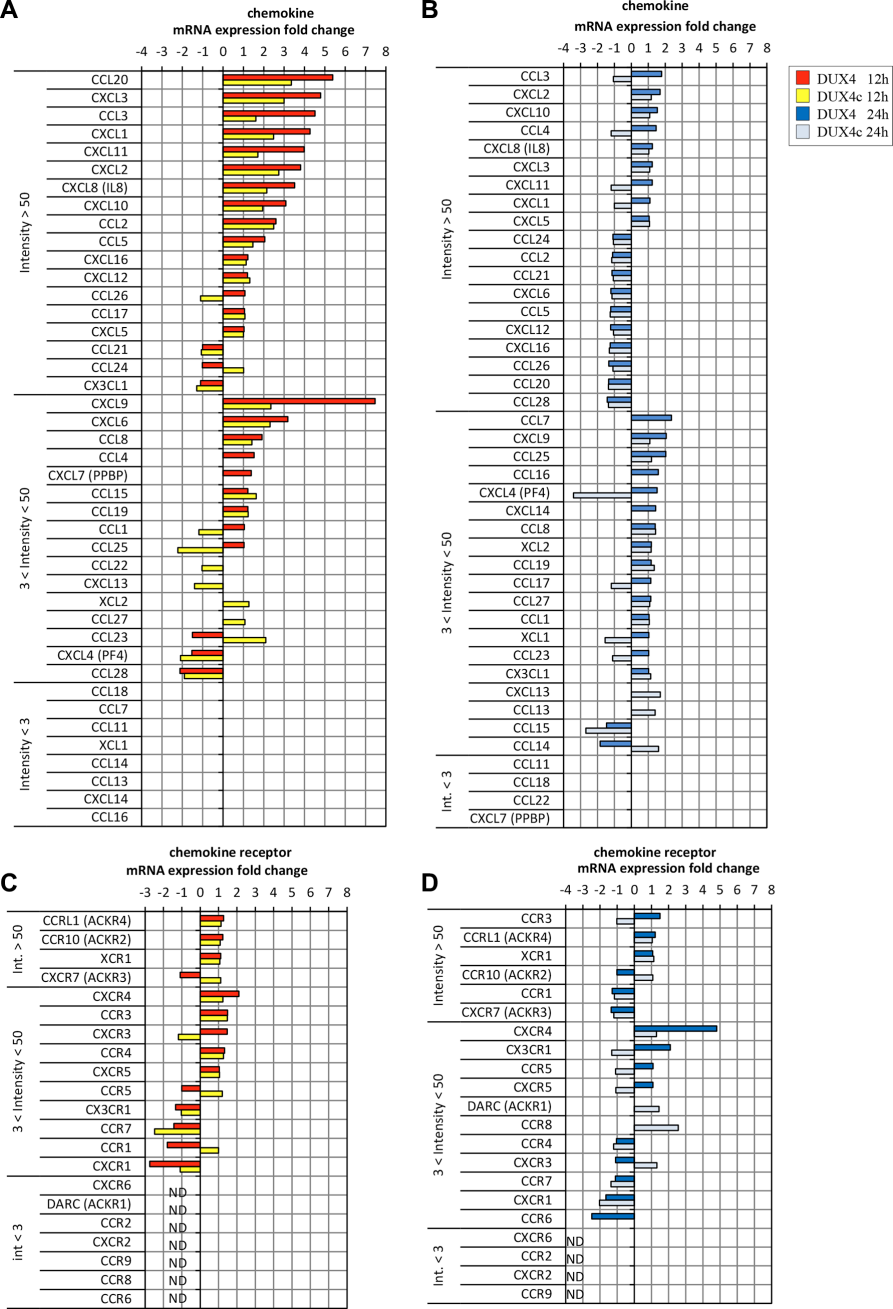
Chemokine (A, B) or chemokine receptor (C, D) gene mRNA expression (microarray) in human immortalized myoblasts (MB) 12 h or 24 h after transfection with DUX4 (A, C) or DUX4c (B, D) plasmids Gene names are divided in 3 classes: highly expressed (int > 50), low expression (3 < int < 50) and non-detectable (in < 3) sorted according to fold change.

**Figure 4:**
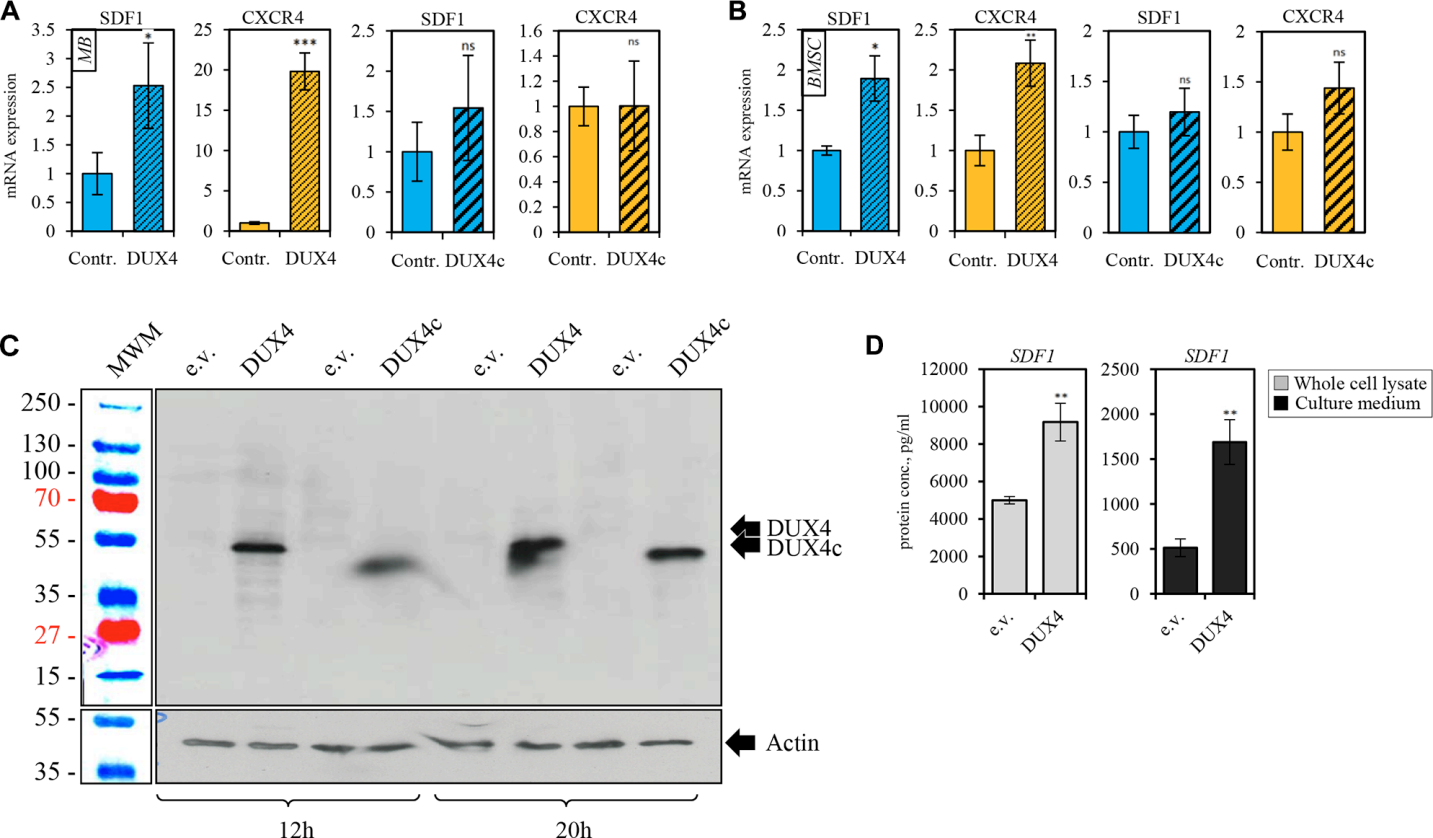
DUX4-transfected cells overexpress SDF1 and CXCR4 genes qRT-PCR analysis of the expression of CXCR4 and SDF1 mRNA in human immortalized myoblasts (MB) (**A**) and bone marrow mesenchymal stem cells (BSMC) (**B**) transiently transfected with DUX4- or, DUX4c-expressing plasmids or an empty vector (pCI-Neo). Average of three independent experiments is shown, error bar represent standard deviation (SD), *t-test p-value* < 0.05 (*). Gene expression level of the control sample normalized to GAPDH was set to 1. (**C**) Western blot analysis of protein lysates of human immortalized myoblasts (MB) transfected with DUX4 and DUX4c plasmids for 12 and 20 h. DUX4 (52-kDa) and DUX4c (47-kDa) proteins were stained with 9A12 antibody. (**D**) SDF1 protein concentration was measured using ELISA in the whole cell lysate (diluted 10-fold) or cell culture medium (concentrated 20 times) of human immortalized myoblasts (MB) 24 h after transfection with pCI-Neo-DUX4, or pCI-Neo (e.v.) plasmids. Average and standard deviation of 3 independent experiments are shown, *t-test p-value* < 0.05 (*).

**Figure 5:**
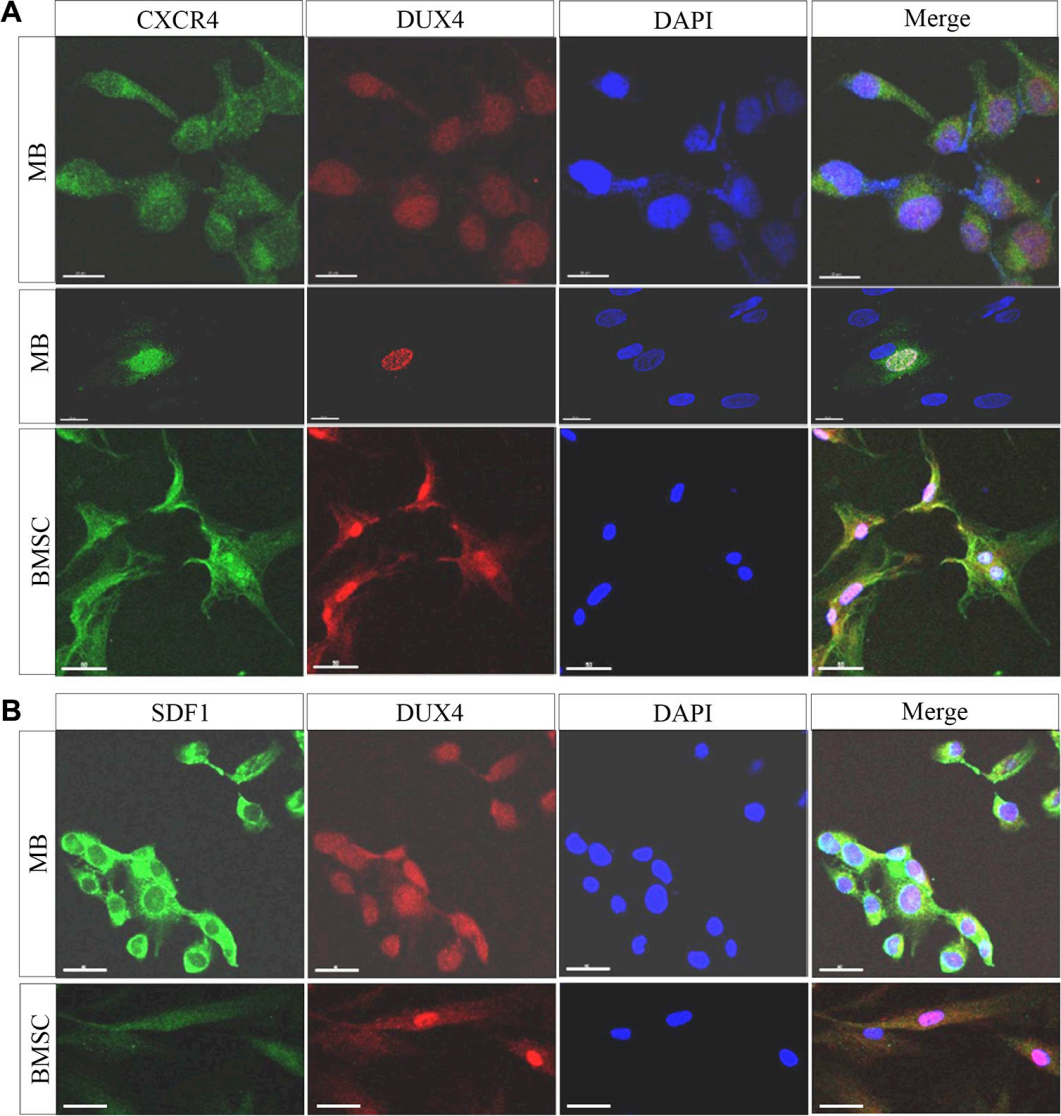
DUX4-transfected cells overexpress SDF1 and CXCR4 Immunofluorescence analysis of CXCR4 and DUX4 expression in human immortalized myoblasts (MB) (**A**) and bone marrow-derived mesenchymal stromal cells (BMSCs) (**B**) 24 h after the transfection with pCI-NeoDUX4 plasmid. Formaldehyde-fixed cells were stained with CXCR4, SDF1 and DUX4 antibodies, representative images are shown; scale bar 50 μm.

DUX4 and DUX4c transcriptome signatures contained genes encoding chemokines. Of 18 chemokine genes reliably detected by the microarray (intensity > 50) in MB, 10 and 7 were at least 1.5-fold upregulated in *DUX4*- and *DUX4c*-overexpressing MB, respectively (Figure 3A, 3B). A relatively weakly expressed *CXCR4* chemokine receptor gene was upregulated 2 to 5-fold in *DUX4-* but not *DUX4c*-overexpressing MB as compared to the controls. Other chemokine receptor genes were either undetectable or not differentially expressed. qPCR and immunofluorescence microscopy analysis of CXCR4 expression in MB and mesenchymal stromal cells isolated from human bone marrow cells (BMSC) transfected with the *DUX4* plasmid confirmed the results of transcriptome profiling (Figures 4, 5 and Supplementary Figure S3). Furthermore, we observed a significant upregulation of *CXCR4* expression in several other cell types transfected with *DUX4* including human keratinocyte cell line HaCat, dermal fibroblasts Dfb and mesenchymal stromal cells isolated from adipose tissue (ADAS) (data not shown).

CXCR4 receptor is activated by the interaction with its only known ligand SDF1. Our microarray analysis did not detect a differential expression of *SDF1* gene (Figure 3C, 3D); however, we observed a moderate but statistically significant upregulation of SDF1 mRNA and protein in *DUX4*- but not *DUX4c*-transfected MB (Figure 4A, 4B). *DUX4*-transfected myoblasts also secreted more SDF1 protein into extracellular medium (Figure 4D). SDF1 is also known to bind to a scavenger receptor CXCR7, however, no change in CXCR7 expression level was observed upon *DUX4* or *DUX4c* overexpression (Figure 3C, 3D). It is also unlikely that *DUX4* expression is essential for *CXCR4* or *SDF1* expression as these genes are known to be expressed in various cells types, and *DUX4* expression is confined to embryonic and germinal cells [17, 11].

### DUX4 overexpression stimulates migration of mesenchymal stem cells

**Figure 6:**
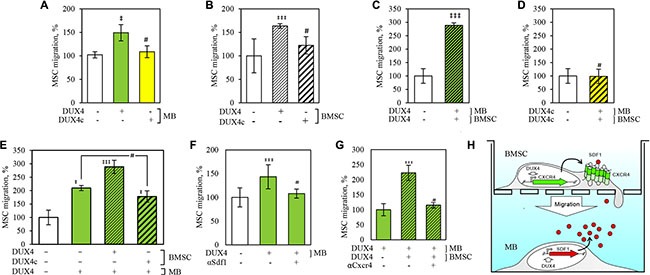
DUX4 and DUX4c expression increases migration rate of human bone marrow stem cells (BMSC) towards human immortalized myoblasts (MB) in a transwell assay BMSC and MB cells were plated to the upper- and the lower wells of a Transwell chamber respectively and the number of BMSC that crossed the membrane and adhered to its lower surface was quantified. (**A**) Migration of non-transfected BMSCs towards MB transfected with DUX4-, DUX4c-plasmid or an empty-vector control (e.v.); (**B**) Migration of BMSCs transfected with DUX4-, DUX4c-plasmid or e.v. towards non-transfected MB; (**C**) Migration of BMSCs transfected with DUX4- or e.v. towards DUX4- or e.v.-transfected MB; (**D**) Migration of BMSCs transfected with DUX4c- or e.v. towards DUX4c- or e.v.-transfected MB; (**E**) Migration of BMSCs transfected with DUX4-, DUX4c- or e.v. towards DUX4- or e.v.-transfected MB; (**F**) Effect of SDF1 antibody on BMSC migration towards MB transfected with DUX4-plasmid or e.v. (**G**) Effect of CXCR4 antibody on migration of BMSC transfected with DUX4-plasmid or e.v. towards DUX4-plasmid or e.v.-transfected MB; (**H**) Schematic representation of DUX4 overexpression impact on BMSC cell migration towards MB. (*), *p*-value < 0.05 as compared to control; #, non-significant; migration to e.v.-transfected cells was set to 100%, error bars correspond to SD.

The efficiency of CXCR4 signaling depends on the expression level of both *SDF1* and *CXCR4* genes (reviewed in [41]. Our results thus suggested that DUX4 might regulate cell mobility and migration. To test the migration and chemoattractive properties of cells overexpressing *DUX4* and *DUX4c*, we used a Transwell assay. Migrating BMSC were plated on an upper layer of a permeable membrane placed in a Transwell insert and chemoattractant-producing human myoblasts were plated to the lower chamber section of the Transwell system (Figure 6H). To test the effect of *DUX4-* and *DUX4c* overexpression on chemoattractive properties of MB, we transfected them with *DUX4* and *DUX4c* plasmids and counted the number of untransfected stromal cells derived from human bone marrow cells (BMSC) that crossed the membrane (Figure 6A). We observed a more efficient migration of BMSC towards *DUX4-* but not *DUX4c* overexpressing MB. The effect was completely blocked if antibodies against SDF1 were added to the culture medium (Figure 6F). We have also tested whether *DUX4* overexpression could increase chemoattractive properties of other cell types. We overexpressed *DUX4* in TE671 rhabdomyosarcoma cell line, known to be resistant to high levels of *DUX4* expression [13, 51], human immortalized myoblasts (MB) and immortalized keratinocyte cells line HaCat and found that *DUX4* overexpression also increases the chemoattractivity of these cells to BMSC (data not shown).

Next, we tested whether *DUX4* or *DUX4c* overexpression could modify migration properties of BMSC, and observed an increase of migration rate of *DUX4-* but not *DUX4c-*trasfected BMSC towards untransfected MB (Figure 6B). This effect was further increased if *DUX4* but not *DUX4c* was simultaneously overexpressed in both BMSC and MB (Figure 6C, 6D). Adding CXCR4 antibodies to the culture medium completely abolished the effect of DUX4 overexpression on cell migration (Figure 6G). Cell migration experiments have been carried out 24 h after the transfection as apoptosis and necrosis rate of the transfected cells at this time point was not significantly different from non-transfected controls (Supplementary Figure S1) arguing against the possibility that cytotoxic effect of DUX4 overexpression could introduce a bias in our assay.

To rule out the possibility that *DUX4* overexpression could affect BMSC and ADAS multipotency, we then induced adipogenic differentiation of *DUX4*-transfected BMSC. We found the number of resulting adipocytes unchanged as compared to a control plasmid-transfected BMSCs, thus arguing against the possibility that *DUX4* altered adipogenic differentiation potential of BMSC and ADAS (Supplementary Figure S2).

We conclude that *DUX4* but not *DUX4c* overexpression increases chemoattractivity of several cell types for MSC migration and also increased migration rate of BMSC when overexpressed in them. The difference in transcriptome signatures of *DUX4* and *DUX4c*-overexpressing myoblasts is thus clearly translated into different biological roles of these transcription factors.

## DISCUSSION

### DUX4 and SDF1-CXCR4 axis in the normal organism

DUX4 is a powerful transcriptional regulator with an unknown physiological role in normal cells. *In vitro*, full-length *DUX4* is expressed in human embryonic and mesenchymal stem cells [17] and induced pluripotent stem cells [11]; its expression is downregulated in the process of differentiation of these cells. DUX4 expression cannot be detected in fully-differentiated cells such as primary myoblasts or fibroblasts where only DUX4-s, a shorter non-toxic form of DUX4 resulting from an alternative splicing of DUX4 gene could be detected.

In normal adult human tissues, full-length DUX4 is expressed only in testis; in somatic tissues including skeletal muscles, liver and heart only DUX4-s transcript was detectable [11]. The pattern of full-length DUX4 expression is indicative of a role at early stages of human development. However, besides the observation that DUX4 overexpression induced neuroectodermal program in murine embryonic stem cells, developmental functions of DUX4 remain unknown. Linking DUX4 with cellular migration described here may thus contribute to the understanding of a physiological role of DUX4 in development.

In the course of vertebrate development, SDF1 and CXCR4 are essential for colonization of the bone marrow by hematopoietic stem and progenitor cells (HSPCs) (reviewed in [52, 53, 39, 38]), colonization of gonads by primordial germ cells (PGCs) [54] (reviewed in [38]); neurogenesis (reviewed in [38, 55]); cardiogenesis and vascular formation [56]; limb myogenesis [57–61]. It is tempting to speculate that as an activator of CXCR4 and SDF1 expression, DUX4 might be also involved in these processes.

The role of DUX4 in development could, in principle, be addressed using loss of function mouse models. Mouse genome contains *DUX4* homologs *Duxbl* (also named *Duxl*) [62, 63] and *Dux*. The degree of amino acid identity of Duxbl and DUX4 reaches 65% in the most conserved region (second homeobox DNA binding domain) [62]. Expression pattern of Duxbl (ovary, eyes, testes and brain of adult mice and developing muscles of mouse embryos [64]) demonstrates a certain similarity to the DUX4 expression pattern. Functional analysis of Duxbl demonstrated that *Duxbl* overexpression increased myoblast proliferation but repressed their myogenic differentiation [65] which to certain extent recapitulated the effect of *DUX4*overexpression on myogenic differentiation.

Another mouse homolog, Dux is only 60% identical to DUX4 in its most conserved region; its expression pattern is also less similar to that of DUX4. Dux expression was demonstrated in adult mouse brain, heart liver and lungs and was almost undetectable in skeletal muscle [62]; functional analysis of this gene was not conducted. Duxbl or Dux loss-of-function mouse models are not yet available, and their relevance for understanding the physiological role is not clear.

### DUX4 and SDF1-CXCR4 axis in pathology

While in normal adult organism the role of DUX4 is unknown and might be limited to germline, the role of DUX4 in pathology (i.e. in Facioscapulohumeral dystrophy or FSHD) has been extensively explored. In this disease, DUX4 expression could be detected in muscle cells and tissues [11] and is thought to contribute to the pathological phenotype by inducing apoptosis, provoking the sensitivity to oxidative stress and inhibiting myogenic differentiation. Inhibition of myogenic differentiation is one of the best understood functions of DUX4 in FSHD. Murine muscle satellite cells expressing human DUX4 are unable to properly differentiate *in vitro* [21] or regenerate skeletal muscle tissue *in vivo* [66].

Inhibition of the myogenic program by DUX4 could be explained by a similarity of recognition sites of DUX4 and Pax3/7 transcription factors essential for the commitment of myogenic progenitors resulting in an interference with the early myogenic differentiation steps [14]. It has also been demonstrated that DUX4 induced the expression of muscular atrophy-related genes [67, 16] myogenic microRNAs [68] and transcription factors [14, 47] suggesting that DUX4 might also interfere with later stages of myogenic differentiation.

Interestingly, CXCR4 is also involved in the regulation of myogenic differentiation. CXCR4 is expressed on the surface of both proliferating and differentiated C2C12 cells [58, 61, 69]. SDF1 expression is increased while CXCR4 decreased during myogenic differentiation of rat myoblasts [70]; however, the role of CXCR4-SDF1 axis in myogenic differentiation remains controversial. Some reports have shown inhibition of myogenic differentiation by SDF1 of CXCR4 signaling [69] or no effect [70]. Other reports have shown that SDF1 induced myotube formation while CXCR4 inhibition via siRNA blocked C2C12 differentiation [71]. The mechanism of CXCR4-dependent regulation of myogenic differentiation is thought to involve MAPK signaling. Intriguingly, MAPK signaling-related genes were identified in DUX4 transcriptomic signature [47]. CXCR4 as one of DUX4 target genes might thus also contribute to FSHD etiology.

Besides an abnormal myogenic differentiation, an accumulation of leukocytes has been also documented in FSHD muscles [72]; the mechanism of this phenomenon is currently unknown. It has been demonstrated that CXCR4 receptor plays a key role in chronic inflammatory conditions such as inflammatory bowel disease (reviewed in [73]), chronic allergic lung inflammation, idiopathic pulmonary fibrosis, liver fibrosis [74], rheumatoid arthritis (reviewed in [75]).

Our results allow us to put forward a hypothesis that DUX4 overexpression resulting in SDF1 expression in FSHD muscles might result in an increased migration of leukocytes to FSHD muscles. It is plausible therefore that DUX4 might be involved in these processes as its aberrant expression in FSHD muscles might activate SDF1 and thus attract circulating CXCR4+ leukocytes which would result in their infiltration to FSHD muscles. Again, this possibility could, in principle, be tested using mouse models of ectopic expression of human DUX4 that have recently been described. Mice expressing human DUX4 under the control of its natural promoter did not show visible phenotypic anomalies except for the eye keratitis of unknown etiology [21]. At the same time, mice expressing DUX4 under the control of a leaky doxycycline-inducible promoter in retina, testis, skin, brain, kidney, and lung, had scaly skin, muscle weakening, increased retina neovascularization, males demonstrated a defect in gametogenesis [66]. Although the latter mouse model had some phenotypical features common to FSHD, its relevance as a model of FSHD is thus unclear.

### DUX4 in cancer

Finally, DUX4 is also known to be expressed in many cancer cell lines including rhabdomyosarcoma RD (CCL-136) and RMS13 (CRL-2061) [13, 76], cervix carcinoma HeLa, lung adenocarcinoma A549 cell lines [76] and in non-small cell lung cancer (NSCLC) cells [76]; DUX4 or a homologous transcript was detected in cervical carcinoma cell line C33A [77]. Recurrent DUX4 fusions have been detected in B cell acute lymphoblastic anemia [78], embryonal rhabdomyosarcoma [79], Ewing-like sarcomas [80] and pediatric primitive round cell sarcomas [81]. The mechanism of DUX4 upregulation in cancer cells remains unknown, although it has been proposed that it is linked to demethylation of the 3.3 kb repeats harboring DUX4 ORF [82, 77, 83].

Tumor cells are known to secrete SDF, inflammatory cytokines and growth factors which together promote MSC homing to the tumor site [84]. MSC infiltration has been proven beneficial for some tumor types [85]. One possibility that is suggested by our results but has not been tested in this study is that DUX4 might contribute to the MSC infiltration into tumors. Another possibility that was not addressed in the present study is that DUX4 expression might influence metastatic potential of cancer cells via CXCR4-SDF1 axis. SDF1-CXCR4 signaling is also involved in migration of cancer cells to the sites of metastasis (SDF1-expressing tissues, such as bone marrow and lung) including breast, lung, ovarian, thyroid, rhabdomyosarcoma and others (over 20 human tumor types (reviewed in [86]) [53, 87, 86–89]. Our study thus prompts to test whether DUX4 overexpressing tumors demonstrate an increased metastatic potential as compared to DUX4-negative tumors.

## MATERIALS AND METHODS

### Cell culture conditions, plasmids and transfection

Human immortalized myoblasts (MB) generated from a healthy subject (a kind gift of Dr. V. Mouly, Institute of Myology, Paris) were cultured in the media composed of 4 parts of high-glucose DMEM, 1 part of Medium 199 (Sigma #M4530) and supplemented with 20% FBS 4 mM L-glutamine 50 mkg/ml gentamicin, 1 mkg/ml Amphotericin B, 2.5 ng/ml human recombinant HGF (Sigma #H1404) and 1 mkM dexamethasone (Sigma #D4902) as described previously [90].

Human primary myoblasts were isolated from skeletal muscle biopsies of healthy subjects and purified using CD56/NCAM magnetic beads (Miltenyi Biotec) as previously described [91], preserved in liquid nitrogen and transported for future use. After unfreezing, the myoblasts were cultured in proliferation medium (high-glucose DMEM (here and below #D6546, Sigma), 20% FBS FBS (Millerium #BWSTS1810/500), 4 mM L-glutamine (Sigma #68540–25 G), 50 μg/ml gentamicin (G1397 Sigma), 1 mkg/ml Amphotericin B (Fungizone, Gibco #15290–018)), passaged at a cell confluence not exceeding 30 % and used for transfection and tests up to passage 5 or earlier to avoid cellular senescence and spontaneous differentiation.

Both primary and immortalized myoblasts were cultured in cell culture dishes coated with collagen using sterile 0.1% solution of collagen powder (#C7661 Sigma) in 0.2% acetic acid.

Human bone marrow-derived mesenchymal cells (BMSC) were isolated as previously described [92]. Briefly, mononuclear cells were isolated from bone marrow aspirates from healthy donors using centrifugation in Histopaque-1077 density gradient (Sigma) at 400 g for 30 min at room temperature; the resulting cells were washed 3 times with DMEM, plated at 10^6 cells/cm2 in cell culture flasks (Greiner Bio-One) and cultured the growth medium (low-glucose DMEM (Gibco) supplemented with 10% fetal bovine serum (FBS) (HyClone), 2 mM GlutaMAX^TM^ (Gibco), and 50 U/ml of penicillin and 50 ug/ml of streptomycin (Gibco); medium was changed every 3 days.

Human adult adipose tissue-derived stem cells (ADAS) were isolated as previously described [92]. Briefly, the adipose tissue aspirates was washed with phosphate-buffered saline (PBS), containing 50 U/ml of penicillin and 50 ug/ml of streptomycin (Gibco), then digested at 37°C for 60 min with 0.075% collagenase type I (Worthington). The cell suspension was washed with DMEM, containing 10% FBS and centrifuged at 300 *g* for 10 min. The pellet was resuspended in 160 mM NH4Cl and incubated at room temperature for 10 min to lyse contaminating red blood cells. The cell suspension was centrifuged as detailed above, the pellet was resuspended in DMEM supplemented with 20% FCS filtered through a 100-um nylon mesh to remove cellular debris, plated at culture flasks and incubated overnight at 37°C, 5% CO2. Following incubation, the flasks were washed extensively with PBS to remove residual nonadherent blood cells. ADAS were cultured in growth medium DMEM/F12 (Gibco), supplemented with 10% FBS, 2 mM GlutaMAX^TM^, 50 U/ml of penicillin and 50 ug/ml of streptomycin (Gibco). The medium was changed every 3 days, cells were maintained at subconfluent levels.

TE-671 and HaCat cell lines were cultured in DMEM medium supplemented with 4.5 g/L glucose (Gibco), 2 mM L-glutamine, 10% fetal bovine serum and 100 u/ml penicillin and 100 ug/ml of streptomycin.

### Adipogenic differentiation

ADAS and BMSC were plated in 12-well plates at a density of 5 × 10^3^ cells cm^-2^ and transfected with DUX4-, DUX4c- or control vector when indicated. After 24 h the growth media was replaced with adipogenic induction medium: DMEM, 10% FBS, 0.5 mM isobutyl-methylxantine (Sigma), 1 uM dexamethasone (Sigma), 10 uM insulin (Sigma), 0.2 mM indomethacin (Sigma); the medium was changed every three days. After 21 days cells were fixed with 4% paraformaldehyde in PBS and lipid droplets were revealed by histochemical staining with Oil Red O dye (Sigma) for neutral fats. For quantification, Oil Red O stain was extracted with 100% isopropanol for 5 min, absorbance was measured at 492 nm and normalized to the number of cells; 100% isopropanol was used as a background control; the experiments were performed in quadruplicates.

Transfection. 8 × 10^4^ of human MB cells were resuspended in 400 mkl of corresponding culturing medium, mixed with 80 mkl of OptiMEM (Gibco #31985062) containing 240 ng of plasmid DNA and 0.24 mkl Lipofectamine 2000 (Invitrogen, #11668–019) and plated into 1 well of a 12-well plate (2.1 × 10^4^ cells/cm2); medium was changed 6 h after the transfection. The transfection efficiency was around 40%. 2 × 10^4^ BMSC or ADAS cells were plated in a well of a12-well plate (5 × 10^3^ cells/cm2) and transfected using 1 μg of plasmid DNA and 1 mkl of Lipofectamine 2000^™^ (Invitrogen) according to the supplier's instructions; medium was changed 6 h after the transfection. The transfection efficiency was 80–90%.

To overexpress DUX4 and DUX4c, we used pCI-Neo-DUX4 [3] and pCI-Neo-DUX4c [22] plasmids containing human DUX4 and DUX4c ORFs genes under control of the CMV promoter (a kind gift of Alexandra Belayew and Frederique Coppée, University of Mons, Belgium); Following control plasmids were used: pCI-Neo (Promega), phrGFP (Stratagene) and pCI-Neo-DUX1 [2].

### Immunofluorescence staining

24 h after the transfection MB or BMSC cells were fixed with 2% PFA (Euromedex) in PBS for 5 min, permeabilized with 0.5% triton X-100 (Sigma-Aldrich) in PBS for 5 min, blocked with 5% BSA (Euromedex) in PBS for 1 h, incubated with mouse monoclonal antibodies against DUX4 clone 9A12 [67] (kindly donated by Alexandra Belayew, University of Mons, Belgium) diluted 1:50; rabbit polyclonal antibody against CXCR4 (Abcam #ab 2074) diluted 1:100 or rabbit polyclonal antibody against SDF1 (CXCL12) (Abcam #ab 9797) diluted 1:200 in 2.5% PBS in BSA, stained with Alexa Fluor 488-conjugated anti-rabbit IgG (Life Technologies #A-21441, 1:100) for 2 h or Alexa Fluor 488 anti-mouse IgG (Life Technologies #A-21200, 1:100) for 1 h 2.5% PBS in BSA and mounted with a mounting medium containing DAPI (Vector laboratories), observed under a fluorescent microscope (Microvision instruments, excitation/emission: 488/519 nm, green fluorescence);

### Western blot

Whole cell protein extracts were prepared from frozen immortalized or primary myoblast cell pellets using TENT buffer (150 mM NaCl, 1 mM EDTA, 50 mM Tris-HCl pH7.5, 0.5% NP-40) [93] supplemented with anti-protease and anti-phosphatase inhibitor cocktails (#04693159001, #04906845001, Roche), separated on 8% PAAG, transferred to a nitrocellulose membrane (Amersham), blocked with 5% milk/PyTBST, hybridized with primary antibodies 9A12 [67] recognizing DUX4 and DUX4c diluted 1:2000, washed with 1 × PyTBST (10 mM Tris-HCl pH7.4, 75 mM NaCl, 1 mM EDTA, 0.1% Tween 20) [94] to reduce background, hybridized with secondary antibodies conjugated to HRP (SantaCruz #sc2030, #sc2768, #sc2005) diluted 1.2000, revealed with ECL+ (GE Healthcare), exposed to X-ray film (Amersham) and developed in a chemical film processor (FujiFilm). Quantification of scanned X-ray images was performed using ImageJ. After exposition the membranes were stripped in a solution containing 2%SDS and 100 mM beta-mercaptoethanol buffered with 62.5 mM TrisHCl, reblocked in 5% BSA/PBS overnight and rehybridized with mouse monoclonal antibodies against actin (Millipore #mab1501) diluted 1:10 000.

Flow cytometry apoptosis assay was performed using a CF™488A Annexin V and 7-AAD Apoptosis Kit (Biotium, cat # 30060). 24 or 72 h after transfection, MB or BMSC cells were trypsinized, centrifuged, resuspended in PBS. To avoid the loss dead cells, the media with detached cells was also collected and centrifuged at 300 g for 5 min, the cells pellet was resuspended in PBS. The cells were stained according manufacture protocol. Briefly, cells were resuspended at 5 × 10^6^ cells/ml in 1X Annexin Binding Buffer, 5 ul of Annexin V and 2 ul of 7-AAD working solutions were added into the cell suspension and incubated at 4°C for 30 min in a dark. Then 400 ul of 1X Annexin Binding Buffer was added and probes were analyzed by flow cytometry on the Cell Lab Quanta SC (Beckman Coulter, Brea, CA, USA)

### Transcriptome profiling

RNA from human MB cells 12- and 20 h after the transfection with pCI-Neo-DUX4, pCI-Neo-DUX4c or pCI-Neo plasmids was extracted using Trizol (Invitrogen) as instructed by the producer and further purified with silica column cleanup using Nucleospin RNA Extraction kit (Macherey Nagel). 500 ng of RNA were used to synthesize Cy3- (control) and Cy5- (DUX4- and DUX4c-transfected MB) labeled probes in a two-step procedure using Agilent Fluorescent Low Input Linear Amplification kit. Mix. Labeled probes were then hybridized to Gene Expression microarrays (4 × 44 k #G4112F, Agilent) and scanned using Agilent G2505C DNA Microarray scanner as instructed by the manufacturer. Scanned images were then analysed using the Feature Extraction software (Agilent, version 10.5.1.1) and gene expression fold change was calculated using Rosetta Resolver (version 7.2.2.0). In the case of DUX4-transfected cells, genes with FC < -3 and Int1 (Cy3) > 50 were considered downregulated; genes with FC > 3 and Int2 (Cy5) > 50 were considered upregulated; in the case of DUX4c-transfected cells, genes with FC < -2 and Int1 (Cy3) > 50 were considered downregulated; genes with FC > 2 and Int2 (Cy5) > 50 were considered upregulated. Gene symbols and descriptions corresponding to significant Agilent IDs were retrieved from the Agilent microarray annotation file and db2db and DAVID gene ID conversion tools. In case of disagreement between the sources, HGNC database (http://www.genenames.org/) was consulted. Probes corresponding to non-annotated genes were not analyzed. Protein-coding genes differentially expressed in DUX4 and DUX4c transfected MB were used for functional annotation with DAVID (http://david.abcc.ncifcrf.gov/) using GOTERM_BP_FAT list and the default background. The stringency of functional annotation clustering was set to “medium”. Clusters with similar biological functions containing at least one significant GO term (acceptable significance: *p-value* < 0.05, FDR < 20) were manually combined using Microsoft Excel with Ablebits add-ons “Cell merge” (https://www.ablebits.com/) resulting is superclusters “Cell cycle”, “Apoptosis” etc.; The significance of a supercluster was considered to be equal to *p-value* and FDR corresponding to the most significant GO term within this supercluster.

### RT-PCR

Total RNA was extracted using Trizol (Invitrogen) as instructed by the producer; the traces of phenol and salts were eliminated with 2 supplementary chlorophorm extractions, ethanol precipitation and two additional washes with 70% ethanol. 100 ng of purified RNA was then reverse transcribed using Revertaid H minus Reverse transcriptase (Fermentas #EP0451) as instructed by the producer, random hexamers and other RT components were also from Fermentas. cDNA was then diluted 10 times and 2 mkl was mixed with primers (300 nM final) FastStart Universal SYBR Green Master mix (ROX) (Roche #04913850001) in a final volume of 20 mkl and analyzed on Step One plus instrument (Applied Biosystems). The sequence of primers (Invitrogen) used: SDF1-F2 5′-GAACGCCAAGGTCGTGGTCGT; SDF1-R2 5′-TCTGTAGCTCAGGCTGACGGGC; CXCR4-F1 5′-A AAGTACCAGTTTGCCACGGC; CXCR4-R1 5′-GCATG ACGGACAAGTACAGGCT; GAPDH-F2 5′-TCATTTC CTGGTATGACAACGA; GAPDH-R2 5′-TACATGGCA ACTGTGAGGAG; Reactions were performed in triplicates, DDct method was used to analyze data [95].

### Migration assays

Migration assays were carried out in a 24-well transwell system equipped with porous (8 μm) polycarbonate membranes. 2 × 10^5^ MB cells were resuspended in 600 μL of growth medium supplemented with 1% FBS and plated into the lower chamber of the transwell system; 5.0 × 10^4^ BMSC cells were resuspended in 200 mkl of growth medium supplemented with 1% FBS and plated into Transwell inserts which were then placed into another transwell system with the lower chamber filled with 600 μl of serum-free medium. After 24 hours the cells on the inserts or in lower chambers were transfected as indicated, 6 hours after the transfection the media was changed and the inserts with BMSC, were placed into the wells with MB cells and incubated at 37 °C for 24 h. When indicated, CXCR4 (Abcam, #ab10403) or SDF1 (CXCL12) (Abcam, #ab9797) antibodies were added to the medium at 10 mkg/ml and 4 mkg/ml concentrations respectively. The inserts were then discarded, and upper sides of the filters were swabbed to remove the cells that did not cross the membrane. The cells present on the lower side of the filters were then fixed in 4 % paraformaldehyde, stained with DAPI and the counted under the microscope. All the experiments were performed in duplicate. The following control antibodies were used: rabbit IgG control (AB-105-C, R&D systems) and mouse IgG2b isotype control (MAB004, R&D systems).

### SDF-1α ELISA assay

3 × 10^6^ MB cells were plated on 10 cm dish and after 24 h were transfected with 8 μg of plasmid DNA using JetPEI as instructed by the producer (Polyplus transfection), ratio plasmid:JetPEI was 1:2; medium was changed 24 h after the transfection. To quantify secreted CXCL12, the medium was collected 48 h after the transfection, centrifuged at 10 000 rpm for 10 min and concentrated 20 times using a 15 ml spin concentrators (5 KDa MWCO) (Agilent Technologies). To quantify intracellular CXCL12, whole cell lysates were prepared in 500 μl of RIPA buffer and diluted 10 times prior to analysis. Samples were analyzed in triplicates using human SDF 1α ELISA Kit (Abcam #ab100637).

## SUPPLEMENTARY MATERIALS FIGURES AND TABLES






