# Supplementary material for: Dux4 controls migration of mesenchymal stem cells through the Cxcr4-Sdf1 axis
Source: Oncotarget. 2016 Aug 18;7(40):65090–108. doi: 10.18632/oncotarget.11368 (PMC5323140; doi:10.18632/oncotarget.11368)
Supplement: Supplementary file 1 [file oncotarget-07-65090-s001.pdf]

Dux4 controls migration of mesenchymal stem cells through the Cxcr4-Sdf1 axis

Supplementary Materials

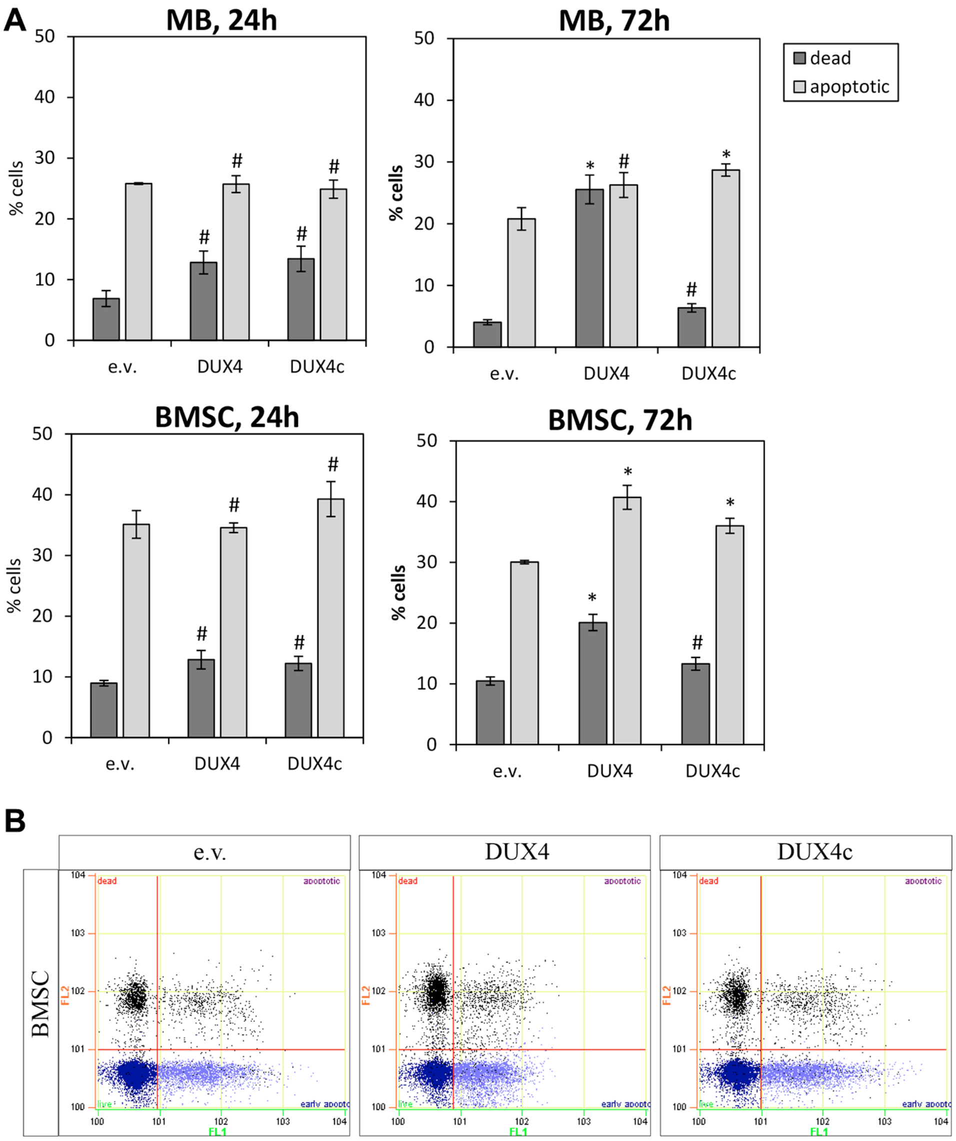

Supplementary Figure S1: (**A**) Results of flow cytometry analysis of apoptosis and cell viability of MB and BMSC cells 24- and 72 h after transfection with DUX4-, DUX4c-plasmid or an empty vector-control. * indicates *t*-test *p*-value < 0.05 for the comparison with the e.v. control; # - no statistical difference as compared to the e.v. control. (**B**) Scatter plot representation of flow cytometry analysis of BMSC cells 72 h after the transfection with DUX4-, DUX4c-plamsid or empty vector-control; FL1: Propidium iodide, FL2: Annexin V.

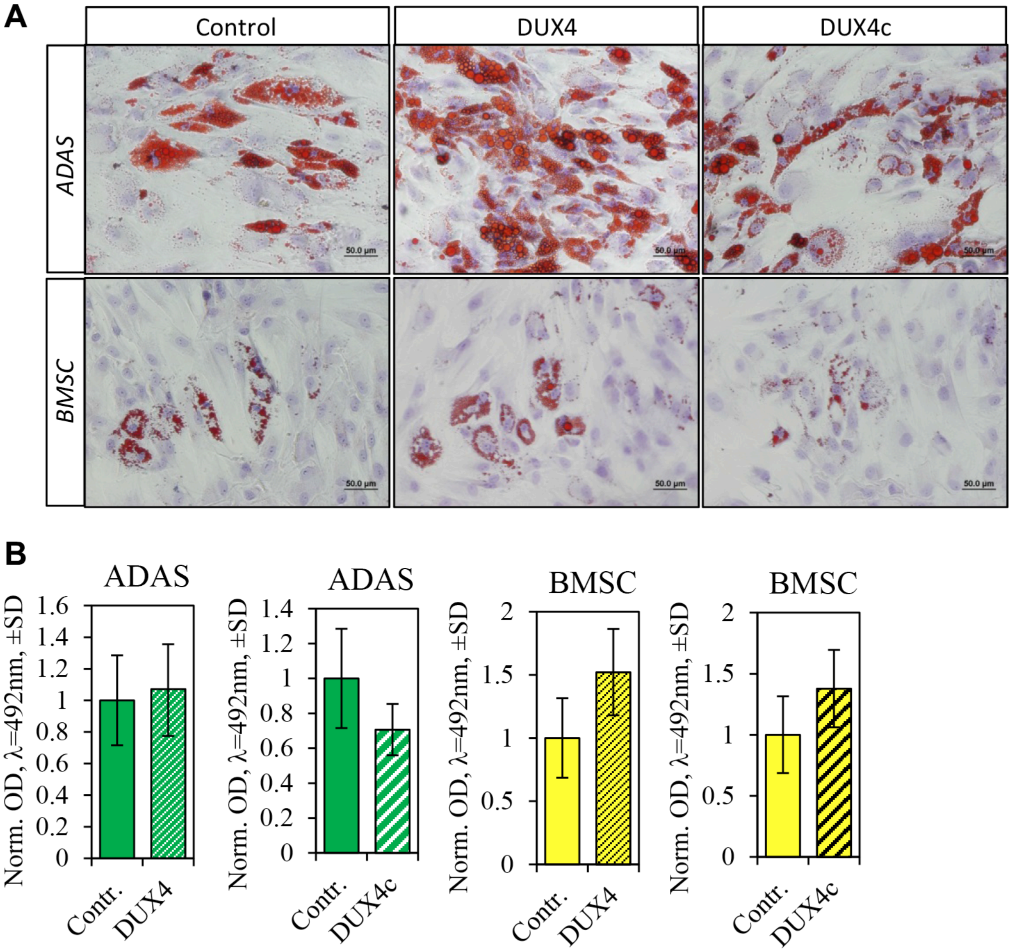

Supplementary Figure S2: DUX4 and DUX4c overexpression does not influence adipogenic differentiation of MSC. (**A**) BMSC or ADAS cells were transiently transfected with DUX4- DUX4c- or control plasmid; adipogenic differentiation was induced 24 h after the transfection; after 21 days of adipogenic differentiation, lipids were stained with Oil Red O and cultures were photographed in phase contrast. (**B**) Results of lipid content quantification at OD 492 nm of BMSC or ADAS cells transfected and differentiated as in (A).

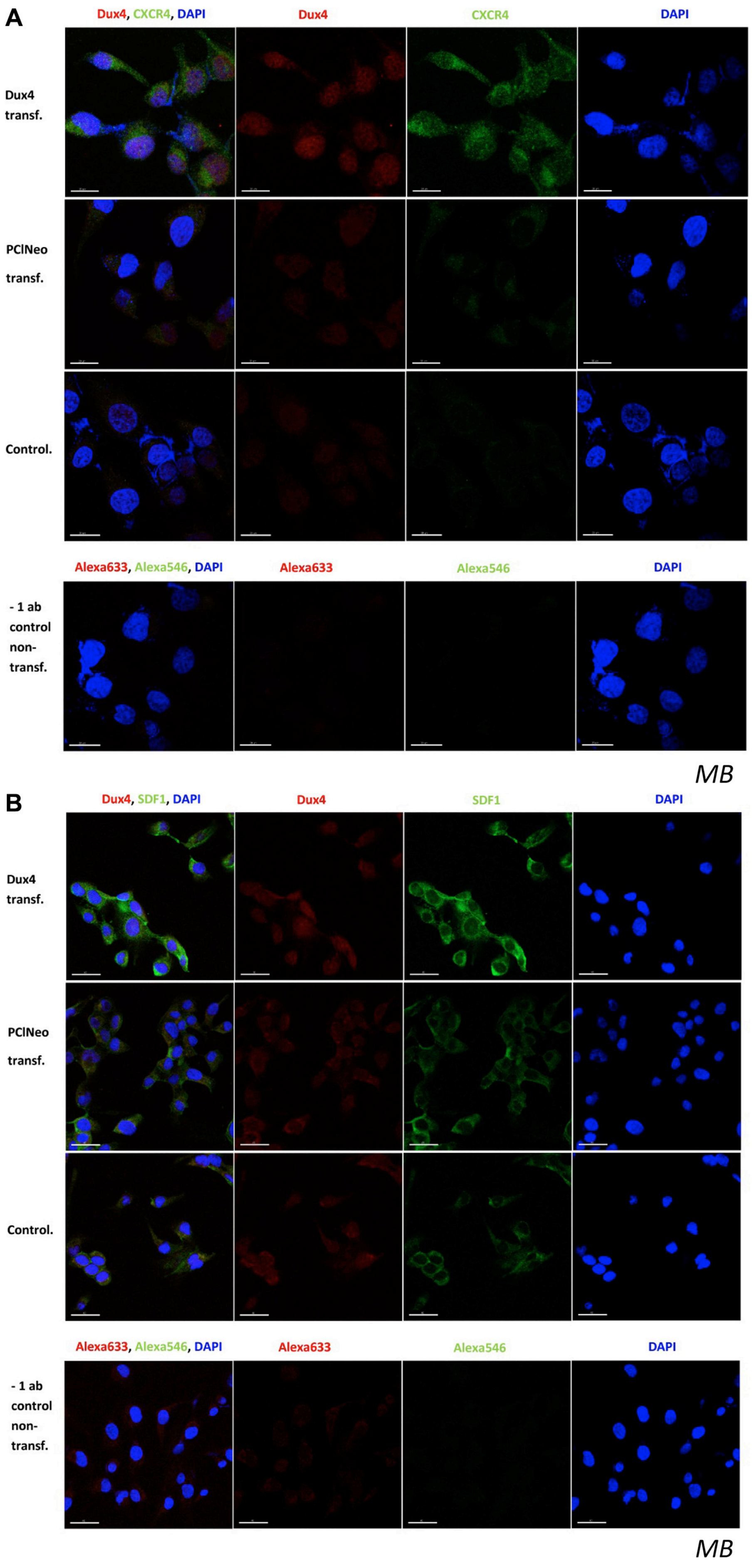
A
Dux4, CXCR4, DAPI
Dux4
CXCR4
DAPI
Dux4
transf.
PCINeo
transf.
Control.
Alexa633, Alexa546, DAPI
Alexa633
Alexa546
DAPI
- 1 ab
control
non-
transf.
MB
B
Dux4, SDF1, DAPI
Dux4
SDF1
DAPI
Dux4
transf.
PCINeo
transf.
Control.
Alexa633, Alexa546, DAPI
Alexa633
Alexa546
DAPI
- 1 ab
control
non-
transf.
MB

C

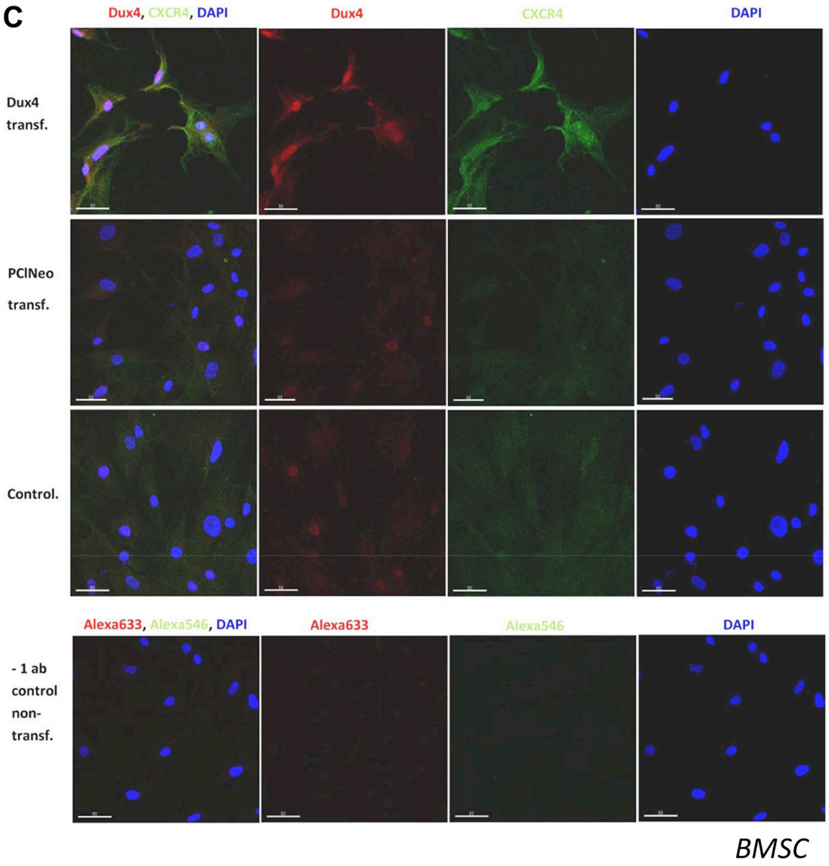

D

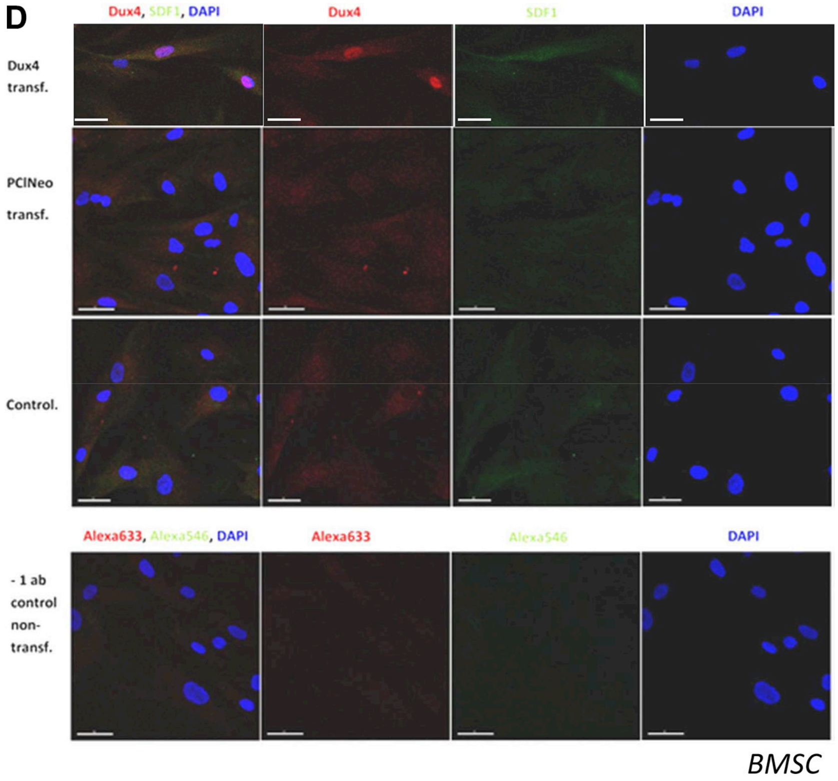

Supplementary Figure S3: DUX4-transfected human immortalized myoblasts (MB) and human bone marrow mesenchymal stem cells (BMSC) overexpress SDF1 and CXCR4. (**A**) Immunofluorescence analysis of CXCR4 and DUX4 expression in MB transfected with pCI-NeoDUX4 plasmid, empty vector (pCI-Neo) or untransfected MB (Control). A no-first-antibody control is shown in the lower row. (**B**) Immunofluorescence analysis of SDF1 and DUX4 expression in MB transfected with pCI-NeoDUX4 plasmid, empty vector or untransfected MB. A no-first-antibody control is shown in the lower row. (**C**) Immunofluorescence analysis of CXCR4 and DUX4 expression in BMSC transfected with pCI-NeoDUX4 plasmid, empty vector or untransfected BMSC. A no-first-antibody control is shown in the lower row. (**D**) Immunofluorescence analysis of SDF1 and DUX4 expression in BMSC transfected with pCI-NeoDUX4 plasmid, empty vector or untransfected BMSC. A no-first-antibody control is shown in the lower row; scale bar = 50 µm.

Supplementary Table S1: Lists of genes differentially expressed in DUX4- or DUX4c- transfected human immortalized myoblasts at two time points (12 h and 20 h) after transfection. See Supplementary_Table_S1

Supplementary Table S2: Functional classification of genes differentially expressed in DUX4- or DUX4c- transfected human immortalized myoblasts at two time points (12 h and 20 h) after transfection; *p*-values and FDR are shown for the top GO term (in bold); Significant superclusters are in bold italic. See Supplementary_Table_S2
