# Supplementary material for: Dux4 controls migration of mesenchymal stem cells through the Cxcr4-Sdf1 axis
Source: Oncotarget. 2016 Aug 18;7(40):65090–108. doi: 10.18632/oncotarget.11368 (PMC5323140; doi:10.18632/oncotarget.11368)
Supplement: Supplementary file 3 [file oncotarget-07-65090-s003.doc]

**Supplementary table S2**

**Table S2.** Functional classification of genes differentially expressed in DUX4- or DUX4c- transfected human immortalized myoblasts at two time points (12h and 20h) after transfection; p-values and FDR are shown for the top GO term (in bold); Significant superclusters are in bold italic.

| Superclass | Transfected | p-value | FDR | DUX4 or DUX4c-specific genes | DUX4 and DUX4c common genes | GO Terms |
| --- | --- | --- | --- | --- | --- | --- |
| ***Inflammation*** | DUX4 | 4.13505E-10 | 6.46471E-07 | CCL3; CCL3L3; CSF3; CXCL9; DHRS2; EGR1; HBEGF | AOC3; CCL2; CCL20; CFB; TNFAIP6; PTX3; SERPINA3; FOS; CXCL1; CXCL10; CXCL11; CXCL2; CXCL3; DEFB119 | **GO:0006954~inflammatory response;** GO:0045087~innate immune response; GO:0006952~defense response; GO:0006955~immune response; GO:0006954~inflammatory response; GO:0009611~response to wounding; GO:0002521~leukocyte differentiation; GO:0030097~hemopoiesis; GO:0048534~hemopoietic or lymphoid organ development; GO:0002520~immune system development |
|  | DUX4c | 1.04737E-09 | 1.59465E-06 | CFI; COLEC12; IL18R1 |  |  |
| ***Chemotaxis/cell motility*** | DUX4 | 3.28268E-07 | 0.000513212 | APOE; CCL3; CCL3L3; CXCL9; HBEGF | CCL2; CCL20; CXCL1; CXCL10; CXCL11; CXCL2; CXCL3; PTGDS | **GO:0042330~taxis;** GO:0006935~chemotaxis; GO:0007626~locomotory behavior; GO:0006928~cell motion; GO:0048870~cell motility; GO:0051674~localization of cell; GO:0007610~behavior; GO:0009611~response to wounding; GO:0006954~inflammatory response; GO:0006952~defense response; GO:0030334~regulation of cell migration; GO:0040012~regulation of locomotion; GO:0051270~regulation of cell motion; GO:0016477~cell migration |
|  | DUX4c | 6.69042E-05 | 0.101814773 | MTSS1; TNP2 |  |  |
| ***Metabolism*** | DUX4 | 0.001433319 | 2.217494338 | EGR1; PLA2G5; | ABCA1; APOC1; APOE; FOXO1; KLF15; NR4A3; FOS; PTX3; YAP1 | **GO:0033700~phospholipid efflux;** GO:0065005~protein-lipid complex assembly; GO:0034377~plasma lipoprotein particle assembly; GO:0033344~cholesterol efflux; GO:0015914~phospholipid transport; GO:0015918~sterol transport; GO:0030301~cholesterol transport; GO:0042157~lipoprotein metabolic process; GO:0008202~steroid metabolic process; GO:0008203~cholesterol metabolic process; GO:0016125~sterol metabolic process; GO:0006869~lipid transport; GO:0010876~lipid localization; GO:0051173~positive regulation of nitrogen compound metabolic process; GO:0031328~positive regulation of cellular biosynthetic process; GO:0009891~positive regulation of biosynthetic process; GO:0045935~positive regulation of nucleobase. nucleoside. nucleotide and nucleic acid metabolic process; GO:0010604~positive regulation of macromolecule metabolic process; GO:0009890~negative regulation of biosynthetic process; GO:0051172~negative regulation of nitrogen compound metabolic process; GO:0031327~negative regulation of cellular biosynthetic process; GO:0045934~negative regulation of nucleobase. nucleoside. nucleotide and nucleic acid metabolic process; GO:0010558~negative regulation of macromolecule biosynthetic process; GO:0010605~negative regulation of macromolecule metabolic process; GO:0046486~glycerolipid metabolic process |
|  | DUX4c | 0.000822798 | 1.245423225 | BCL7A; CDC6; CYP21A2; EGR1 |  |  |
| ***Apoptosis*** | DUX4 | 0.016302736 | 22.66136316 | DHRS2; SIAH1; TNFSF14 | APOE; CCL2; FOXO1; HGF; PIM3; TNFAIP3; ZC3H12A | **GO:0043066~negative regulation of apoptosis ;** GO:0006916~anti-apoptosis; GO:0043069~negative regulation of programmed cell death; GO:0060548~negative regulation of cell death; GO:0042981~regulation of apoptosis; GO:0043067~regulation of programmed cell death; GO:0010941~regulation of cell death; GO:0006915~apoptosis; GO:0012501~programmed cell death; GO:0008219~cell death; GO:0016265~death |
|  | DUX4c | 0.012378117 | 17.27395789 | HIP1; TRAF1 |  |  |
| Ion homeostasis | DUX4 | 0.088929130345184 | 76.6847163725739 | CACNA1F; CCL3; KCNAB2; SLC34A2; TNFSF14 | ABCA1; APOE; CCL2 | **GO:0006873~cellular ion homeostasis;** GO:0055082~cellular chemical homeostasis; GO:0050801~ion homeostasis; GO:0042592~homeostatic process; GO:0019725~cellular homeostasis; GO:0048878~chemical homeostasis; GO:0030001~metal ion transport; GO:0006812~cation transport; GO:0006811~ion transport; GO:0006874~cellular calcium ion homeostasis; GO:0055074~calcium ion homeostasis; GO:0006875~cellular metal ion homeostasis; GO:0055065~metal ion homeostasis; GO:0030005~cellular di-. tri-valent inorganic cation homeostasis; GO:0055066~di-. tri-valent inorganic cation homeostasis; GO:0030003~cellular cation homeostasis; GO:0055080~cation homeostasis |
|  | DUX4c | 0.22123367406091 | 97.1328595379442 | none |  |  |
| Transcription | DUX4 | 0.0678509733230637 | 66.6622992600998 | FOXP2; LEUTX; MYB; TNRC6C; ZIM3; ZSCAN4 | ABCA1; APOC1; APOE; BCL7A; DUX3 EGR1 FOS; FOXO1 HMGA2 HPX-2; KLF15; NFIL3 NR4A3; YAP1 | **GO:0045893~positive regulation of transcription. DNA-dependent;** GO:0051254~positive regulation of RNA metabolic process; GO:0045944~positive regulation of transcription from RNA polymerase II promoter; GO:0045941~positive regulation of transcription; GO:0010628~positive regulation of gene expression; GO:0010557~positive regulation of macromolecule biosynthetic process; GO:0010604~positive regulation of macromolecule metabolic process; GO:0006357~regulation of transcription from RNA polymerase II promoter; GO:0006355~regulation of transcription. DNA-dependent; GO:0051252~regulation of RNA metabolic process; GO:0045449~regulation of transcription; GO:0006350~transcription; GO:0045935~positive regulation of nucleobase. nucleoside. nucleotide and nucleic acid metabolic process; GO:0010629~negative regulation of gene expression; GO:0051172~negative regulation of nitrogen compound metabolic process; GO:0016481~negative regulation of transcription; GO:0045934~negative regulation of nucleobase. nucleoside. nucleotide and nucleic acid metabolic process |
|  | DUX4c | 0.0564271613044152 | 58.6999832999774 | none |  |  |
| Angiogenesis | DUX4 | 0.167814546003134 | 94.3411818038039 | none | APOE; FOXO1; TNFAIP1; ZC3H12A | **GO:0001568~blood vessel development;** GO:0001944~vasculature development; GO:0048514~blood vessel morphogenesis |
|  | DUX4c | 0.0912394574444883 | 76.6985119128639 | none |  |  |
| Neuron development | DUX4 | 0.184891072584748 | 95.9078613333281 | HBEGF | APOE; CCL2; EGR1 | **GO:0050804~regulation of synaptic transmission;** GO:0051969~regulation of transmission of nerve impulse; GO:0031644~regulation of neurological system process; GO:0044057~regulation of system process |
|  | DUX4c | 0.118667861829353 | 85.3870431456101 | none |  |  |
| Cell signaling | DUX4 | 0.19531480325664 | 96.6537201992991 | CACNA1F; CHML; DGKE | APOE; CCL2; CFB; EFNA1; FOS; HGF; PIM3; PRPH2 | **GO:0009991~response to extracellular stimulus;** GO:0007584~response to nutrient; GO:0031667~response to nutrient levels; GO:0043405~regulation of MAP kinase activity; GO:0043085~positive regulation of catalytic activity; GO:0044093~positive regulation of molecular function; GO:0045859~regulation of protein kinase activity; GO:0043549~regulation of kinase activity; GO:0051338~regulation of transferase activity; GO:0042325~regulation of phosphorylation; GO:0019220~regulation of phosphate metabolic process; GO:0051174~regulation of phosphorus metabolic process; GO:0007243~protein kinase cascade; GO:0006468~protein amino acid phosphorylation; GO:0016310~phosphorylation; GO:0006796~phosphate metabolic process; GO:0006793~phosphorus metabolic process; GO:0045860~positive regulation of protein kinase activity; GO:0033674~positive regulation of kinase activity; GO:0051347~positive regulation of transferase activity; GO:0007601~visual perception; GO:0050953~sensory perception of light stimulus; GO:0007600~sensory perception |
|  | DUX4c | 0.0710856133670379 | 67.4596201461561 | CDC6; HIP1; SSTR1 |  |  |
| Cell cycle | DUX4 | 0.363014074910106 | 99.9133483949016 | CCNA1; SIAH1 | HGF; HMGA2 | **GO:0000280~nuclear division;** GO:0007067~mitosis; GO:0000087~M phase of mitotic cell cycle; GO:0048285~organelle fission; GO:0000279~M phase; GO:0000278~mitotic cell cycle; GO:0022403~cell cycle phase; GO:0022402~cell cycle process; GO:0007049~cell cycle |
|  | DUX4c | 0.24916523811825 | 98.7261013331932 | CDC6 |  |  |
| Cellular transport | DUX4 | 0.36301407491010684 | 99.91334839490169 | CCL3; CHML; SNX22 | ABCA1;APOE; CXCL10; NFKBIE; PTX3 | **GO:0006897~endocytosis;** GO:0010324~membrane invagination; GO:0016044~membrane organization; GO:0016192~vesicle-mediated transport; GO:0008104~protein localization; GO:0015031~protein transport; GO:0045184~establishment of protein localization |
|  | DUX4c | 0.0154256521725992 | 21.0763549449329 | COLEC12; HIP1; TNP2 |  |  |
| Protein degradation | DUX4 | 0.40816551127167 | 99.9725468917521 | SIAH1; TRIM63; USP29 | APOE; TNFAIP3 | **GO:0006511~ubiquitin-dependent protein catabolic process;** GO:0019941~modification-dependent protein catabolic process; GO:0043632~modification-dependent macromolecule catabolic process; GO:0051603~proteolysis involved in cellular protein catabolic process; GO:0044257~cellular protein catabolic process; GO:0030163~protein catabolic process; GO:0044265~cellular macromolecule catabolic process; GO:0009057~macromolecule catabolic process; GO:0006508~proteolysis |
|  | DUX4c | - | - | none |  |  |
| Cellular morphology (morphogenesis) | DUX4 | 0.412198104837867 | 99.9753299607649 | CACNA1F; SIAH1 | HGF | **GO:0000904~cell morphogenesis involved in differentiation;** GO:0000902~cell morphogenesis; GO:0032989~cellular component morphogenesis |
|  | DUX4c | - | - |  |  |  |
| Protein complex assembly | DUX4 | - | - | none | none | **GO:0006461~protein complex assembly;** GO:0070271~protein complex biogenesis; GO:0065003~macromolecular complex assembly; GO:0043933~macromolecular complex subunit organization |
|  | DUX4c | 0.0826756652458644 | 65.7211747211887 | COLEC12; HIP1; TRAF1 |  |  |
